# FGFR1 suppresses ovarian cancer progression by modulating SIRT3-dependent lactylation and metabolic reprogramming

**DOI:** 10.1038/s41420-026-03054-6

**Published:** 2026-04-07

**Authors:** Fan Jiang, Huaju Huang, Zhe Dong, Lu Zhang, Shiti Zhang, Mengnan Long, Xue Fan, Nan Li, Hao Ai

**Affiliations:** 1 The Third Affiliated Hospital of Jinzhou Medical University, Jinzhou, China; 2 Jinzhou Medical University, Jinzhou, China; 3 Liaoning Provincial Key Laboratory of Follicle Development and Reproductive Health, Jinzhou, China; 4 Harbin Medical University, Harbin, China; 5 Second Affiliated Hospital of Harbin Medical University, Harbin, China; 6 Jinan University, Guangzhou, China

**Keywords:** Cancer metabolism, Prognostic markers

## Abstract

Ovarian cancer (OC) is an aggressive gynecological malignancy with poor prognosis, largely due to late-stage diagnosis and high metastatic potential. However, the functional role and regulatory mechanisms of fibroblast growth factor receptor 1 (FGFR1) in OC remain incompletely understood. In this study, we investigated the expression pattern and biological function of FGFR1 in OC and explored its underlying molecular mechanisms. FGFR1 expression was analyzed using TCGA, GTEx, and tissue microarray datasets, and its prognostic significance was evaluated by Kaplan–Meier survival analysis. Functional assays were performed in OVCAR-3 and SK-OV-3 cells following FGFR1 knockdown or overexpression to assess cell proliferation, migration, invasion, and metabolic activity, including extracellular acidification rate (ECAR) and oxygen consumption rate (OCR). Lactate production and histone lactylation were measured by biochemical assays and Western blotting. Protein interaction between FGFR1 and SIRT3 was examined by co-immunoprecipitation and immunofluorescence, and rescue experiments were conducted to determine SIRT3 dependency. In vivo subcutaneous xenograft models were used to evaluate the role of FGFR1 in tumor growth. We found that FGFR1 expression was significantly reduced in OC tissues and that low FGFR1 levels were associated with unfavorable clinical outcomes. Functionally, FGFR1 silencing promoted OC cell proliferation, migration, invasion, and metabolic activity, whereas FGFR1 overexpression exerted inhibitory effects. Mechanistically, FGFR1 interacted with SIRT3 and stabilized its protein expression. Importantly, SIRT3 knockdown abrogated the FGFR1-mediated reductions in lactate production, glycolytic enzyme expression, ATP levels, and histone lactylation, indicating that FGFR1 regulates metabolic reprogramming through a SIRT3-dependent mechanism. Consistently, FGFR1 knockdown promoted the formation of larger and more invasive tumors in vivo. Collectively, these findings demonstrate that FGFR1 functions as a context-dependent tumor suppressor in OC by modulating SIRT3-mediated metabolic reprogramming and histone lactylation, suggesting that targeting the FGFR1–SIRT3 axis may represent a potential therapeutic strategy for ovarian cancer.

## Introduction

Among gynecologic cancers, ovarian cancer (OC) ranks as one of the deadliest, primarily due to its late-stage diagnosis and high metastatic potential [1]. Approximately 85–90% of OC originate from epithelial cells, making this subtype the most lethal among gynecologic malignancies [2, 3]. Despite advancements in chemotherapy and targeted therapies, 5-year overall survival remains under 50% [4, 5]. This underscores the urgent need for reliable biomarkers to improve early detection, prognostic assessment, and therapeutic strategies for OC.

Fibroblast growth factor receptor 1 (FGFR1) is a key receptor tyrosine kinase that is often amplified or overexpressed in many tumors [6]. Its activation regulates cell proliferation, differentiation, and metabolic reprogramming through various signaling pathways including PI3K/AKT, RAS/RAF/MEK/ERK, and STATs [7–9]. Recent studies have shown that FGFR1 activation significantly enhances glycolysis [10]. It increases glucose uptake and promotes the expression of important glycolytic enzymes, including HK2, PFK1, and LDHA, which in turn accelerates lactate production [11]. This metabolic change not only provides tumor cells with sufficient energy and building blocks but also helps them adapt to adverse conditions like hypoxia, thereby promoting tumor growth and invasion [12, 13].

At the same time, lactate, as the end product of glycolysis, accumulates in the tumor microenvironment and serves a critical function in cell signaling, immune regulation, and gene expression [14, 15]. Recent discoveries have also identified lactate as a substrate for a novel post-translational modification called lactylation [16, 17]. This new modification may contribute to the regulation of tumor progression. However, no research has yet directly examined the association between FGFR1 signaling and lactylation, especially in OC—a tumor type that shows significant metabolic reprogramming.

In this research, we showed that FGFR1 exhibits low expression levels in OC patients, which is associated with a favorable prognosis. Our results reveal that FGFR1 can restricts OC cell proliferation, migration, and invasion. Moreover, we observed that FGFR1 suppresses lactylation in OC. This finding offers novel insights on the metabolic regulation and microenvironment of OC and lays the theoretical groundwork for future therapeutic strategies targeting FGFR1.

## Results

### FGFR1 is lowly expressed in OC tissues and closely associated with clinical outcomes

**Fig. 1:**
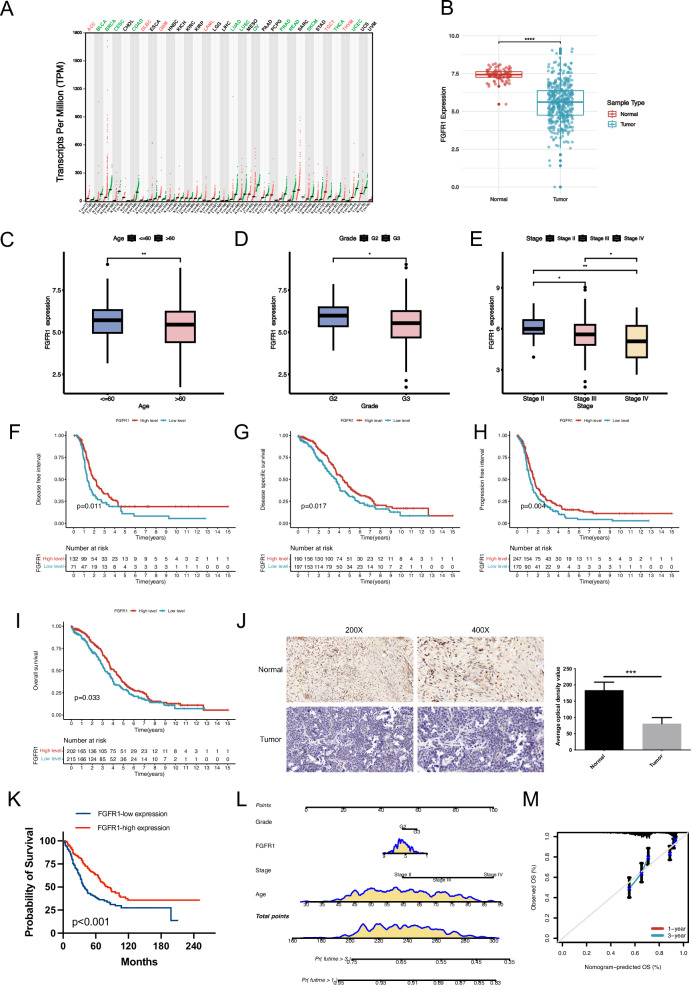
Aberrant FGFR1 expression in OC and its association with prognosis. **A** Pan-cancer analysis reveals abnormal expression of FGFR1 across multiple tumor types. **B** FGFR1 expression is significantly downregulated in OC compared to normal tissues, as demonstrated by TCGA and GTEx datasets. **C–E** FGFR1 expression is lower in patients aged >60 years (**C**), in tumors with a higher histological grade (G3) (**D**), and in advanced-stage tumors (Stage IV) (**E**). **F–I** Survival analysis illustrate that patients with lower FGFR1 expression exhibit notably worse survival outcomes, including DFS (**F**), DSS (**G**), PFS (**H**), and OS (**I**) compared to those with higher FGFR1 expression. **J** IHC staining confirms reduced FGFR1 expression in OC tissues versus normal tissues (200×, 400×), with quantification showing a significant decrease in optical density. **K** Survival analysis indicates that lower FGFR1 expression correlates with poorer prognosis. **L** A nomogram integrating age, pathological grade, clinical stage, and FGFR1 expression level is constructed to predict the prognosis of OC patients. **M** The calibration curves confirm the accuracy of the nomogram in predicting 1-year and 3-year survival, showing strong consistency between predicted and observed survival rates.

Our pan-cancer analysis showed abnormal FGFR1 expression across multiple tumor types (Fig. 1A). Notably, FGFR1 shows significantly lower expression in OC tissues compared to normal counterparts (Fig. 1B). In addition, FGFR1 levels are significantly associated with clinical features of OC patients. Specifically, lower expression was observed in patients over 60 years old, as well as in those with Grade 3 and Stage IV (Fig. 1C-E). Survival analysis revealed that FGFR1 is significantly associated with disease-free interval (DFI), progression-free interval (PFI), disease-specific survival (DSS), and overall survival (OS) in OC patients (Fig. 1F–I). Lower levels of FGFR1 expression corresponded to poorer patient outcomes. Immunohistochemical analysis of OC and normal tissues confirmed the low expression of FGFR1 in tumor samples (Fig. 1J), and further demonstrated that reduced FGFR1 expression is significantly associated with poor prognosis in OC patients (Fig. 1K). Based on these findings, we Constructed a nomogram to predict patient prognosis with high accuracy (Fig. 1L, M). Overall, these findings underscore the valuable contribution of FGFR1 in the progression of OC.

### Transcriptomic alterations and immune features linked to FGFR1 in OC

**Fig. 2:**
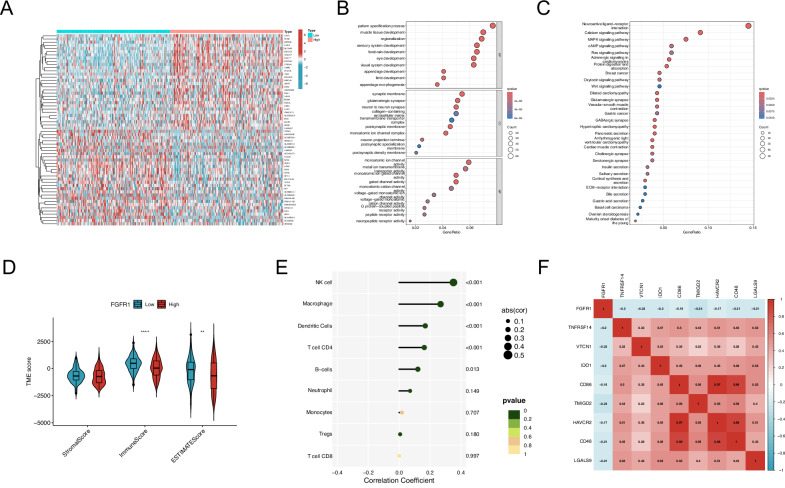
Pathway enrichment and immune correlation analysis of FGFR1 in OC. **A** Heatmap displays the top 30 DEGs between the high and low FGFR1 expression groups. **B**, **C** GO (**B**) and KEGG (**C**) enrichment analyses of the DEGs, illustrating the biological processes, molecular functions, and pathways significantly associated with FGFR1 expression. **D** Comparison of stromal score, immune score, and ESTIMATE score between the high and low FGFR1 expression groups, indicating significant differences in the tumor microenvironment. **E** Correlation analysis between FGFR1 expression and the abundance of different immune cell types, demonstrating significant associations with macrophages, NK cells, dendritic cells, and CD4^+^ T cells. **F** Heatmap displaying the correlation between FGFR1 and immune checkpoint molecules.

We divided OC patients into high- and low-FGFR1 groups based on median expression and identified 1571 differentially expressed genes (50 downregulated, 1521 upregulated), with the top 30 shown in Fig. 2A. GO enrichment indicated that these genes were mainly associated with muscle tissue development (BP), synaptic membrane components (CC), and ion channel activity (MF) (Fig. 2B). KEGG pathway analysis further revealed enrichment in Wnt, Ras, MAPK signaling, and ECM–receptor interaction pathways (Fig. 2C), suggesting that FGFR1 may regulate OC progression through multiple signaling networks.

Immune infiltration analysis showed that patients with low FGFR1 expression had significantly higher immune scores (Fig. 2D). FGFR1 expression was positively correlated with dendritic cells, macrophages, B cells, and CD4⁺ T cells (Fig. 2E), and negatively associated with several immune checkpoints, including TNFRSF14 and VTCN1 (Fig. 2F). These findings indicate that FGFR1 expression is closely linked to immune cell infiltration and checkpoint regulation, suggesting a potential role for FGFR1 in modulating antitumor immune responses.

### FGFR1 modulates the proliferation, migration, and invasion of OC cells

**Fig. 3:**
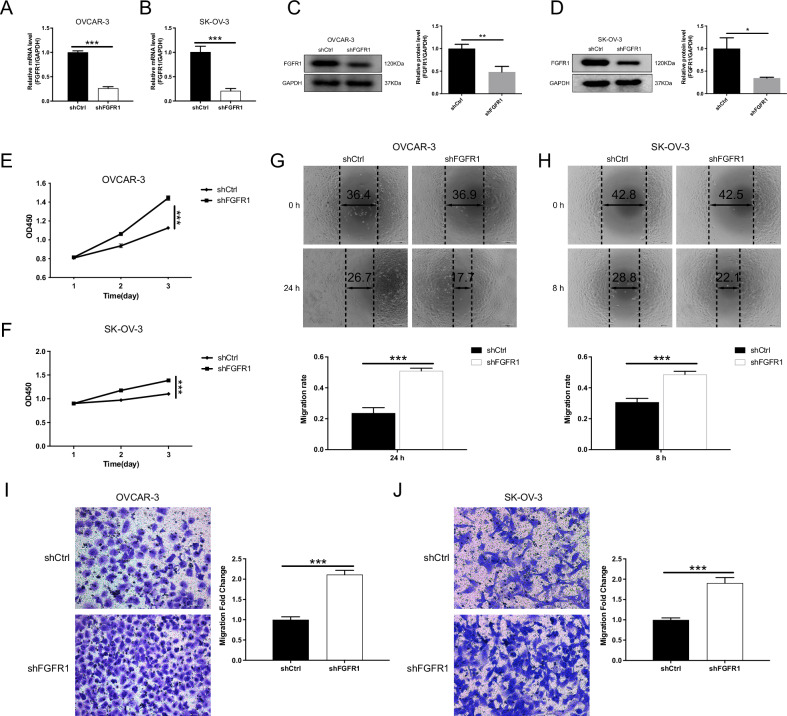
Knockdown of FGFR1 promotes the progression of OC cells. **A–D** Establishment of FGFR1 knockdown cell lines using lentiviral transduction. qPCR (**A**, **B**) and Western blotting (**C**, **D**) confirm the efficiency of FGFR1 knockdown in OVCAR-3 and SK-OV-3 cells. **E**, **F** CCK-8 assays indicate that FGFR1 knockdown significantly enhances the proliferation of OC cells. **G**, **H** Wound healing assays show that FGFR1 knockdown markedly promotes cell migration in OVCAR-3 (**G**, 24 h) and SK-OV-3 (**H**, 8 h) cells. **I**, **J** Transwell invasion assays further demonstrate that FGFR1 knockdown significantly enhances the invasive capability of OVCAR-3 (**I**) and SK-OV-3 (**J**) cells. The data are presented as mean ± SD (*n* = 3). Statistical analysis was performed using the unpaired Student’s t-test for two-group comparisons, *P* values: *p < 0.05, **p < 0.01, ***p < 0.001”.

**Fig. 4:**
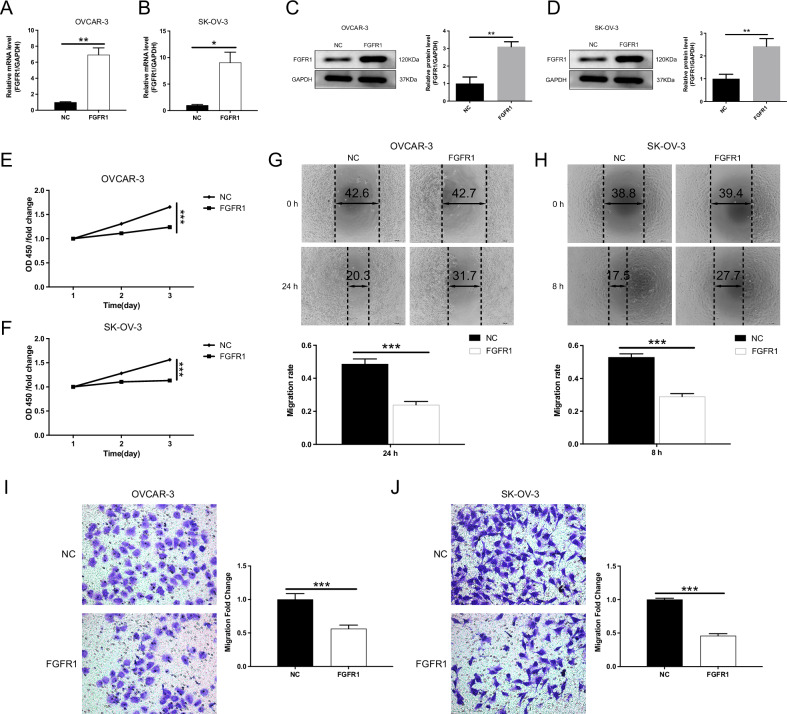
Overexpression of FGFR1 inhibits the progression of OC cells. **A–D** Establishment of FGFR1-overexpressing OC cell lines using lentiviral transduction. qPCR (**A**,**B**) and Western blotting (**C**,**D**) confirm the successful overexpression of FGFR1 in OVCAR-3 and SK-OV-3 cells. **E**,**F** CCK-8 assays show that FGFR1 overexpression significantly suppresses the proliferation of OC cells. **G**,**H** Wound healing assays show that FGFR1 overexpression markedly inhibits cell migration in OVCAR-3 (**G**, 24 h) and SK-OV-3 (**H**, 8 h) cells. **I**,**J** Transwell invasion assays further demonstrate that FGFR1 overexpression significantly reduces the invasive capacity of OVCAR-3 (**I**) and SK-OV-3 (**J**) cells. The data are presented as mean ± SD (*n* = 3). Statistical analysis was performed using the unpaired Student’s t-test for two-group comparisons, *P* values: *p < 0.05, **p < 0.01, ***p < 0.001”.

FGFR1 knockdown in SK-OV-3 and OVAR-3 cells was confirmed by qRT-PCR and Western blot, showing a significant reduction in FGFR1 expression (Fig. 3A–D). Functional assays demonstrated that FGFR1 silencing significantly promoted OC cell aggressiveness. The shFGFR1 group exhibited higher proliferation rates (Fig. 3E, F), increased migration at 8 and 24 h (Fig. 3G, H), and stronger invasion potential (Fig. 3I, J) compared to the control group, highlighting the role of FGFR1 in suppressing these malignant behaviors. On the other hand, FGFR1 overexpression in SK-OV-3 and OVAR-3 cells significantly increased FGFR1 expression, as confirmed by qRT-PCR and Western blot (Fig. 4A–D). Overexpression of FGFR1 reduced cell proliferation (Fig. 4E, F), migration (Fig. 4G, H), and invasion (Fig. 4I, J), indicating that FGFR1 acts as a suppressor of these aggressive behaviors in OC cells.

### FGFR1 influences lactylation modification in ovarian cancer cells

**Fig. 5:**
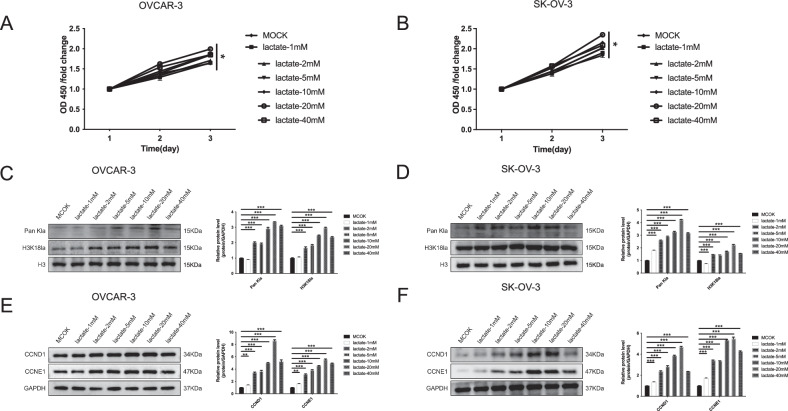
Lactate modulates the progression of OC cells. **A**, **B** CCK-8 assays reveal that increasing concentrations of lactate gradually enhance the viability of OC cells. **C**, **D** Western blot analysis demonstrates that lactate treatment leads to a dose-dependent increase in global protein lactylation (Pan-Kla) and histone H3K18 lactylation (H3K18la) in both OC cells. **E**, **F** Western blot analysis of cell cycle regulators shows that lactate upregulates the expression of CCND1 (Cyclin D1) and CCNE1 (Cyclin E1) in a concentration-dependent manner in OC cells, indicating a potential role of lactate in promoting cell cycle progression. The data are presented as mean ± SD (*n* = 3). Statistical analysis was performed using one-way ANOVA, *P* values: *p < 0.05, **p < 0.01, ***p < 0.001”.

To assess the impact of lactate on OC cells, SK-OV-3 and OVCAR-3 cells were treated with increasing lactate concentrations (1, 2, 5, 10, 20, 40 mM). CCK-8 assays showed a dose-dependent increase in proliferation, accompanied by elevated levels of lactylation-related proteins (Pan-Kla, H3K18la) and the cell-cycle regulators CCND1 and CCNE1 (Fig. 5A–F). Notably, both proliferation and protein expression peaked at 20 mM, indicating that lactate at optimal concentrations promotes OC cell growth and histone lactylation.

**Fig. 6:**
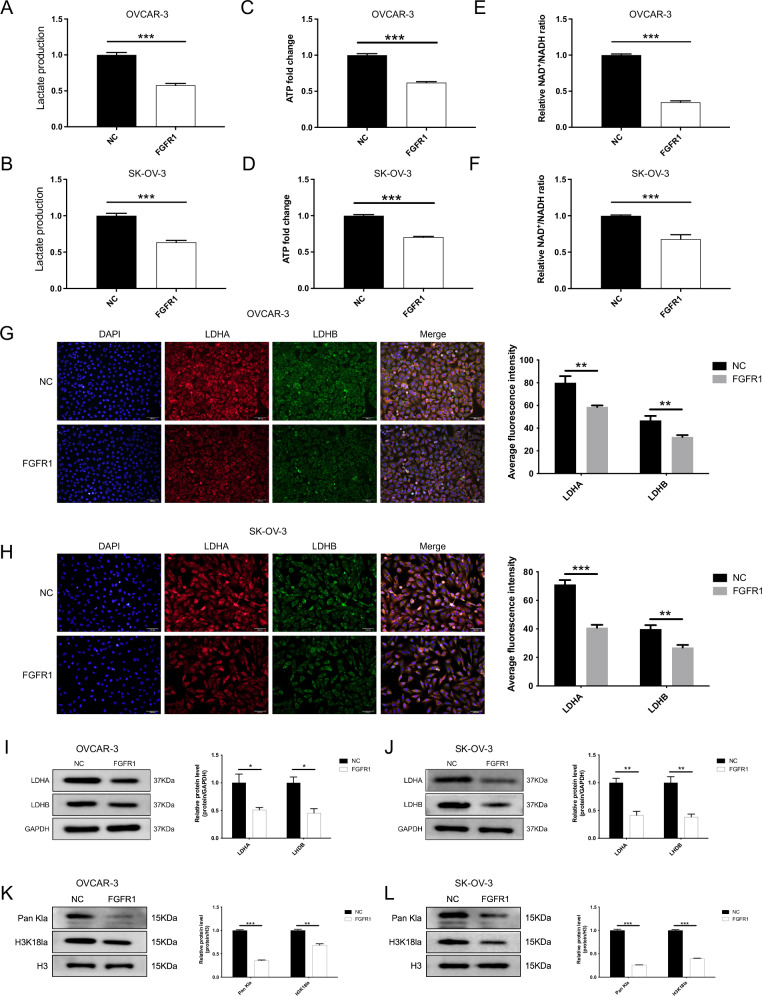
FGFR1 Regulates lactate metabolism and lactylation in OC cells. **A**, **B** Overexpression of FGFR1 significantly reduces lactate production in OC cells. **C**, **D** ATP levels are significantly decreased upon FGFR1 overexpression in OC cells. **E**, **F** NAD + /NADH ratios are significantly reduced in FGFR1-overexpressing OC cells, suggesting altered cellular metabolism. **G**, **H** Immunofluorescence staining shows that FGFR1 overexpression markedly reduces LDHA and LDHB expression in OC cells. **I**, **J** Western blot analysis confirms that FGFR1 overexpression significantly decreases LDHA and LDHB protein levels in both OC cell lines. **K**, **L** Western blot analysis further demonstrates that FGFR1 overexpression significantly reduces Pan-Kla and H3K18la in OC cells. The data are presented as mean ± SD (*n* = 3). Statistical analysis was performed using one-way ANOVA, ns, not significant; *p < 0.05; **p < 0.01; ***p < 0.001.

Comparisons between FGFR1-overexpressing and control cells further revealed that FGFR1 overexpression reduced intracellular lactate levels, the NAD⁺/NADH ratio, and ATP production (Fig. 6A–F). Immunofluorescence and Western blotting confirmed decreased LDHA/LDHB expression and reduced Pan-Kla and H3K18la levels in FGFR1-overexpressing cells (Fig. 6G–L). These findings indicate that FGFR1 suppresses lactate-associated metabolic activity and lactylation, thereby modulating metabolic reprogramming and epigenetic regulation in OC cells.

### FGFR1 regulates SIRT3-dependent metabolic reprogramming in OC cells

**Fig. 7:**
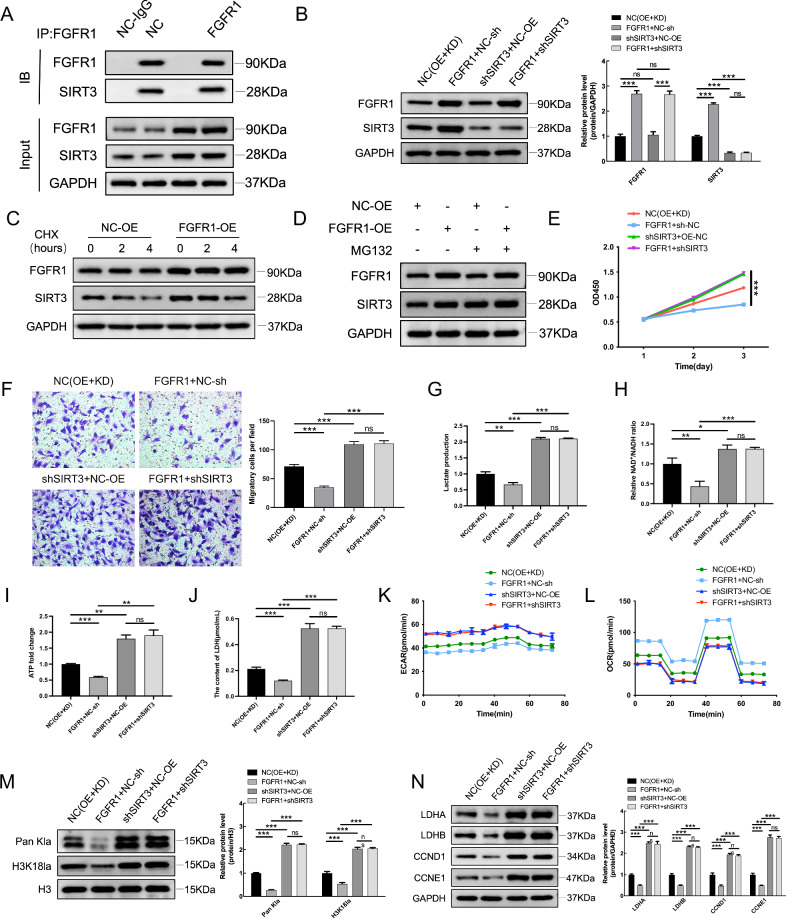
FGFR1 regulates SIRT3-dependent metabolic reprogramming in OC cells. **A** Co-IP analysis confirming the interaction between endogenous FGFR1 and SIRT3. **B** Western blot showing FGFR1 and SIRT3 levels in FGFR1-overexpressing or FGFR1-knockdown cells with or without SIRT3 manipulation. **C** Cycloheximide (CHX) chase assay showing the stability of SIRT3 protein in control and FGFR1-overexpressing cells at the indicated time points. **D** Western blot analysis showing the effects of the proteasome inhibitor MG132 on SIRT3 protein levels in cells with or without FGFR1 overexpression. **E** CCK-8 assay assessing cell proliferation in OC cells with the indicated genetic manipulations. **F** Transwell migration assay evaluating the migratory capacity of OC cells under the indicated conditions. **G** Lactate production in OC cells with FGFR1 and/or SIRT3 manipulation. **H** Intracellular NAD⁺/NADH ratio in the indicated groups. **I** ATP levels measured in OC cells with the indicated treatments. **J** LDH enzymatic activity in OC cells under the indicated conditions. **K** Extracellular acidification rate (ECAR) measured by Seahorse analysis, reflecting glycolytic activity. **L** Oxygen consumption rate (OCR) measured by Seahorse analysis, reflecting mitochondrial oxidative metabolism. **M** Global protein lactylation (Pan Kla) and histone H3K18 lactylation (H3K18la) detected by Western blot analysis. **N** Western blot analysis and quantification of LDHA, LDHB, CCND1, and CCNE1 expression in the indicated groups. Data are shown as mean ± SD. ns, not significant; *p < 0.05; **p < 0.01; ***p < 0.001.

TCGA analysis showed that FGFR1 expression was positively correlated with several metabolic regulatory genes involved in glycolysis and lactate-related pathways, including HK2, PFKM, HDAC2, and SIRT3 (Fig. S1A–D). In line with these findings, qRT-PCR and Western blot analyses demonstrated that FGFR1 overexpression increased the mRNA and protein levels of HK2, PFKM, HDAC2, and particularly SIRT3 (Fig. S1E–F). Immunofluorescence analysis revealed clear intracellular colocalization between FGFR1 and SIRT3 (Fig. S1G). qRT–PCR analyses showed that FGFR1 overexpression significantly increased SIRT3 mRNA levels, whereas knockdown of SIRT3 did not affect FGFR1 mRNA expression (Fig. S1H–I), indicating that FGFR1 functions upstream of SIRT3. Co-immunoprecipitation further confirmed an interaction between FGFR1 and endogenous SIRT3 (Fig. 7A). Although FGFR1 induces SIRT3 transcription, transcriptional upregulation alone does not necessarily ensure sustained protein abundance. We therefore assessed whether FGFR1 additionally regulates SIRT3 at the post-translational level. In cells overexpressing FGFR1, SIRT3 protein abundance was increased compared with controls (Fig. 7B). Cycloheximide (CHX) chase assays further demonstrated that FGFR1 overexpression markedly delayed SIRT3 decay, indicating that FGFR1 prolongs the half-life of SIRT3 (Fig. 7C). Moreover, proteasome inhibition by MG132 restored SIRT3 protein levels under FGFR1-deficient conditions (Fig. 7D), suggesting that FGFR1 maintains SIRT3 stability by attenuating proteasome-dependent degradation. Functionally, FGFR1 overexpression significantly suppressed OC cell proliferation, whereas SIRT3 silencing largely reversed the anti-proliferative effects driven by FGFR1 (Fig. 7E). Similarly, Transwell assays showed that FGFR1 overexpression reduced migratory capacity, which was substantially restored upon SIRT3 depletion (Fig. 7F). These data support that SIRT3 is required for FGFR1-mediated suppression of OC cell growth and motility.

Metabolic analyses showed that FGFR1 overexpression reduced lactate production, the NAD⁺/NADH ratio, and ATP levels, whereas SIRT3 knockdown partially reversed these changes (Fig. 7G–I). Notably, LDH assay further supported that FGFR1 rewires lactate-associated metabolic activity in a SIRT3-dependent manner (Fig. 7J).

To comprehensively characterize metabolic flux, Seahorse profiling was performed. FGFR1 overexpression suppressed glycolytic activity as reflected by decreased extracellular acidification rate (ECAR), while promoting mitochondrial oxidative metabolism as indicated by increased oxygen consumption rate (OCR); both effects were blunted by SIRT3 knockdown (Fig. 7K–L). Consistent with reduced lactate availability, FGFR1 overexpression decreased global protein lactylation, including Pan-Kla and histone H3K18la, whereas SIRT3 depletion restored these lactylation signals (Fig. 7M). Finally, FGFR1 overexpression reduced the expression of lactate metabolism enzymes (LDHA/LDHB) and cell-cycle regulators (CCND1/CCNE1), and these effects were largely abrogated by SIRT3 knockdown (Fig. 7N). Together, these findings demonstrate that FGFR1 regulates OC cell behavior and lactate-associated metabolic reprogramming through a SIRT3-dependent mechanism.

### In vivo validation of FGFR1’s Role in ovarian cancer growth

**Fig. 8:**
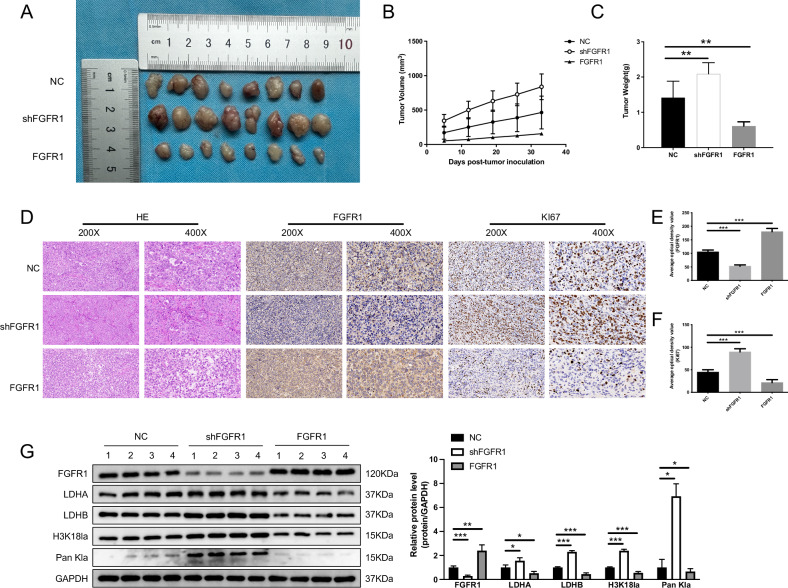
In vivo effects of FGFR1 on tumor growth and lactylation in OC. **A–C** Tumor images (**A**), growth curve (**B**), and tumor weight (**C**) from the xenograft models (*n* = 8). **D–F** HE and IHC staining of tumor sections. HE staining for tumor histopathology, IHC staining for FGFR1 (**E**), and Ki-67 staining for proliferation (**F**). **G** Western blot analysis of tumor tissues. Data are shown as mean ± SD. ns not significant; *p < 0.05; **p < 0.01; ***p < 0.001.

To further assess FGFR1 function in vivo, we conducted subcutaneous xenograft experiments using OVCAR-3 cells in nude mice. We implanted three groups of OVCAR-3 cells into the mice: FGFR1 knockdown, FGFR1 overexpression, and a control group. Results indicated that tumors in the FGFR1 knockdown group were significantly larger than those in the control group, whereas FGFR1 overexpression resulted in notably smaller tumors (Fig. 8A–C). In addition, H&E, Ki67, and FGFR1 immunohistochemical staining further supported these findings. H&E staining revealed a more invasive tumor phenotype in the knockdown group, and Ki67 staining indicated increased cell proliferation (Fig. 8D–F). Western blot analysis confirmed that the expression level of FGFR1 in tumor tissues affected lactate production and lactylation modifications (Fig. 8G). Together, these in vivo results reinforce the critical role of FGFR1 in regulating OC growth and proliferation.

## Discussion

OC is a serious health risk for women and remains one of the leading causes of death among gynecological malignancies [3]. OC is often diagnosed at a late stage because early symptoms are minimal, and its aggressive nature—marked by rapid cell proliferation, invasion, and metastasis—complicates treatment [18]. Furthermore, recurrence and chemotherapy resistance add to the clinical challenges. Recently, molecular targeted therapies have shown promise in overcoming these barriers [19, 20].

FGFR1 plays a vital role in many physiological and pathological processes in various cell types, such as epithelial cells, fibroblasts, endothelial cells, and muscle cells [21, 22]. Normally, when FGFR1 binds its ligand (FGF), it dimerizes and autophosphorylates, triggering downstream pathways that are important for development, cell differentiation, and tissue repair [23–25]. However, abnormal FGFR1 signaling is associated with several cancers. For instance, studies have shown elevated FGFR1 expression in cancers like squamous cell lung cancer and breast cancer, and patients with FGFR1 amplification tend to have worse outcomes [26, 27]. Importantly, these findings do not conflict with our results, as the biological role of FGFR1 is highly context-dependent. Similar to other developmental regulators such as TGF-β [28] and NOTCH [29], FGFR1 may exhibit either oncogenic or tumor-restraining activity depending on cellular lineage, genetic background, ligand availability, and downstream signaling configuration. In contrast to cancers where FGFR1 is amplified, our analyses indicate that FGFR1 is downregulated in OC, and reduced FGFR1 expression consistently correlates with more advanced disease and poor patient survival. Functionally, FGFR1 knockdown enhanced, whereas FGFR1 overexpression suppressed, malignant phenotypes in OC cells and xenograft models. These findings support a context-specific tumor-suppressive role of FGFR1 in OC rather than a universal one.

Tumor cells are well known to undergo metabolic reprogramming characterized by elevated aerobic glycolysis and lactate accumulation [30]. Lactate not only functions as a metabolic byproduct but also acts as an epigenetic regulator by inducing histone lactylation, thereby remodeling chromatin structure and modulating gene transcription [31, 32]. Based on this metabolic–epigenetic link, we hypothesized that FGFR1 might influence histone lactylation through its regulatory effects on glycolytic flux. Indeed, we observed that exogenous lactate stimulation significantly enhanced OC cell proliferation and markedly increased lactylation-related proteins (Pan-Kla, H3K18la), as well as cell cycle regulators (CCND1, CCNE1). Conversely, FGFR1 overexpression reduced intracellular lactate levels, decreased the NAD⁺/NADH ratio, and lowered ATP production. Moreover, FGFR1 overexpression diminished the expression of key glycolytic enzymes (LDHA, LDHB) and suppressed global protein lactylation. These findings indicate that FGFR1 inhibits lactate production and lactylation-driven epigenetic activation, thereby restraining OC cell aggressiveness. Importantly, our mechanistic studies revealed that FGFR1 regulates these metabolic and lactylation-related changes through SIRT3, a mitochondrial deacetylase and critical metabolic regulator [33]. FGFR1 interacts with SIRT3 and stabilizes its protein expression, thereby positioning SIRT3 as a downstream mediator of FGFR1-mediated metabolic regulation. Accordingly, FGFR1-induced metabolic suppression appears to be largely dependent on SIRT3 availability, as SIRT3 knockdown attenuated the inhibitory effects of FGFR1 on lactate production, LDH activity, glycolytic enzyme expression, ATP generation, and histone lactylation. Notably, FGFR1 was also associated with increased SIRT3 mRNA expression, suggesting that FGFR1 may regulate SIRT3 at multiple levels. Transcriptional induction together with post-translational stabilization may cooperatively contribute to maintaining SIRT3 protein abundance and metabolic function in ovarian cancer cells. Although the potential involvement of other SIRT family members cannot be excluded, the present study focused on elucidating the role of SIRT3 in FGFR1-mediated metabolic regulation, based on its established function as a mitochondrial metabolic regulator and our functional evidence showing that SIRT3 depletion consistently attenuated FGFR1-induced metabolic suppression. Collectively, these findings are consistent with a model in which SIRT3 serves as a primary downstream mediator of FGFR1-dependent metabolic reprogramming in ovarian cancer cells.

Given that lactate accumulation promotes immunosuppression and epigenetic remodeling in various tumors [34, 35], the FGFR1–SIRT3–lactate axis may also influence the immune environment in OC. Supporting this possibility, our immune infiltration analysis showed a significant negative correlation between FGFR1 expression and multiple immune checkpoints. Previous studies in head and neck cancer have demonstrated that FGFR-TKIs enhance responses to immune checkpoint inhibitors by increasing MHC class I/II expression via MAPK signaling [36]. Additionally, FGFR1 overexpression in non-small cell lung cancer has been associated with reduced T-cell infiltration and lower PD-L1 expression [37], suggesting a broader immunomodulatory role. Together with our findings, these observations highlight the potential of combining FGFR1-targeted approaches with immunotherapy to counteract immune escape in OC.

Despite the robust findings presented in this study, several limitations must be acknowledged. First, most experiments were conducted using two established OC cell lines (SK-OV-3 and OVCAR-3), which may not fully represent the heterogeneity of different OC subtypes. Although these models provided valuable mechanistic insights, additional studies using a broader panel of OC cell lines or patient-derived models are needed to determine the generalizability of our findings. Second, most in vivo experiments were performed using a subcutaneous xenograft model in nude mice, which does not fully recapitulate the complex tumor microenvironment of human OC. Finally, future investigations incorporating patient-derived xenograft models and larger clinical cohorts will be essential to validate our observations and further evaluate the therapeutic potential of targeting FGFR1 in OC.

## Conclusion

In summary, our study highlights the critical role of FGFR1 in OC. FGFR1 governs not only cell proliferation, migration, and invasion but also influences lactate metabolism and histone lactylation. These findings suggest that targeting FGFR1 could offer a novel therapeutic approach to suppress OC progression and improve patient outcomes.

## Materials and Methods

### Data collection

The transcriptomic profiles of OC tissues and adjacent normal tissues were sourced from the TCGA (https://www.cancer.gov/tcga) and GTEx (https://www.gtexportal.org/home/) databases. The TCGA database contains transcriptomic data and clinical information from 379 OC samples, while the GTEx database provides transcriptomic data from 88 normal ovarian tissue samples. The tissue microarray, comprising samples from 365 OC patients along with corresponding prognostic data, was sourced from the Third Affiliated Hospital of Jinzhou Medical University. All experiments adhered to the ethical guidelines of the Declaration of Helsinki. Prior to sample collection, participants were thoroughly informed about the study and provided written consent. Ethical approval was granted by the Ethics Committee of the Third Affiliated Hospital of Jinzhou Medical University.

### Identification of differentially expressed genes and pathway enrichment analysis

We employed the limma R package to identify differentially expressed genes (DEGs) between FGFR1^high^ and FGFR1^low^ groups. The selection criteria for DEGs included an absolute log fold change ( | logFC | ) greater than 1 and a false discovery rate (FDR) below 0.05. To gain deeper insights into the functional roles and biological pathways potentially associated with FGFR1, we conducted enrichment analyses based on Gene Ontology (GO) and the Kyoto Encyclopedia of Genes and Genomes (KEGG) using the “clusterProfiler” R package, which provides comprehensive tools for gene functional classification and enrichment [38].

### Immune microenvironment analysis

The ESTIMATE algorithm was applied to evaluate stromal and immune cell infiltration in malignant tumors using expression data. This method quantified tumor-infiltrating immune and stromal cell scores across different groups based on gene expression patterns [39]. Immune cell and function enrichment scores for individual sample were determined using the single sample gene set enrichment analysis (ssGSEA) method [40, 41], and their correlation with FGFR1 expression levels were analyzed.

### Cell culture and transfection procedures

Human OC cell lines OVCAR-3 and SK-OV-3 were sourced from the American Type Culture Collection. The cells were cultured in RPMI 1640 medium (Gibco, USA) supplemented with 1% antibiotics (100 μg/mL penicillin G and 100 μg/mL streptomycin, Gibco, USA) and 10% FBS (Gibco, USA) under standard conditions (37 °C, 5%CO₂). Upon reaching 30% confluence, lentiviral vectors for FGFR1 knockdown or overexpression were introduced into serum-free medium at a multiplicity of infection (MOI) of 10. Cells underwent incubation for 16–20 h before the medium was refreshed with complete growth medium. Subsequent assays were conducted 48–72 h post-transduction. Detailed information regarding the lentiviral constructs is provided in Supplementary Table 1.

### Quantitative PCR (qPCR)

Total RNA was extracted from the cells using TRIzol reagent (Invitrogen, USA) and then reverse-transcribed into cDNA with a commercial kit (Thermo, USA). Quantitative PCR was carried out on a Bio-Rad chemiluminescence imager using SYBR Green Master Mix (Thermo, USA), with FGFR1 expression normalized to GAPDH via the 2^-ΔΔCt^ method. The detailed information of the primers is provided in Supplementary Table 2.

### Western blot and co-immunoprecipitation

Cells were lysed in RIPA buffer (Beyotime, China) fortified with protease inhibitors (Beyotime, China), and total protein concentrations were measured via a BCA assay (Beyotime, China). Equal protein amounts were resolved by SDS-PAGE and electrotransferred onto PVDF membranes. The membranes were then blocked for 1 h with 5% nonfat milk before undergoing overnight incubation with primary antibodies. Following extensive washing, HRP-conjugated secondary antibodies were applied, and protein bands were subsequently visualized via enhanced chemiluminescence. For Co-immunoprecipitation (Co-IP) analysis, equal amounts of whole-cell lysates were incubated with Protein A/G magnetic beads (Beyotime, China) and the corresponding primary antibodies at 4 °C overnight. The bead-bound immune complexes were then washed thoroughly, eluted by boiling in 5× SDS loading buffer, and analyzed by Western blotting as described above. Comprehensive details regarding the antibodies can be found in Supplementary Table 3.

### Cell counting kit-8 (CCK-8) assay

The transfected cells were seeded in 96-well plates at 2000 cells/well, and incubated under specific conditions for 24, 36, and 72 h. 10ul of CCK-8 reagent (Beyotime, China) were added to each well and incubated at 37 °C for 2 h, after which absorbance at 450 nm was measured to assess cell viability.

### Wound Healing Assay

Cells (5×10⁴/well) were seeded into 96-well plates and grown to near 100% confluence. A linear scratch was created using a 200-μL pipette tip, followed by three PBS washes to remove debris. Cells were then cultured for an additional 8 or 24 h. Images were captured at 0 h and 8/24 h, and the wound area was quantified using ImageJ. Migration rate was calculated as:

Migration rate (%) = ((migrated area - initial area) / initial area) × 100%.

### Transwell Invasion Assay

Cells (5×10⁴ in 100 μL serum-free medium) were seeded into Matrigel-coated Transwell inserts. The lower chamber was filled with 600 μL medium containing 10% FBS. After incubation at 37 °C with 5% CO₂ for 24 h, invaded cells were fixed with 4% paraformaldehyde for 15 min and stained with crystal violet for 15 min, followed by PBS washing. Non-invading cells on the upper surface were removed. Invaded cells were imaged under a microscope, and cell numbers were quantified in three randomly selected fields.

### Subcutaneous tumor xenograft model

To evaluate the in vivo effects of FGFR1, a subcutaneous xenograft model was established using 4–6-week-old nude mice. OVCAR-3 cells with FGFR1 knockdown, FGFR1 overexpression, or control vectors were injected subcutaneously into the flanks. Tumor growth was monitored every five days using calipers, and volumes were calculated as:

Volume = π/6 × Length × Width².

After one month, mice were euthanized and tumors were harvested, photographed, and measured. For histological analysis, tumors were fixed in 10% formalin, paraffin-embedded, sectioned at 4 μm, and stained with hematoxylin and eosin (H&E). Sections were examined under a light microscope to evaluate morphological alterations.

### Immunohistochemical (IHC) staining

To perform immunohistochemistry (IHC), tumor sections embedded in paraffin were first deparaffinized and rehydrated. Antigen retrieval was carried out by heating the sections in citrate buffer (pH 6.0). Antigen retrieval was performed by heating the sections in citrate buffer (pH 6.0). To suppress endogenous peroxidase activity, the slides were treated with 3% hydrogen peroxide. Subsequently, non-specific binding sites were blocked using 5% bovine serum albumin, followed by overnight incubation with primary antibodies at 4 °C. After washing, HRP-conjugated secondary antibodies were applied, and color development was achieved using a diaminobenzidine substrate. Finally, the sections were counterstained with hematoxylin, mounted, and examined under a microscope.

### Measurement of lactate, NAD⁺/NADH, and ATP levels in OC cells

Lactate, NAD^+^/NADH, and ATP levels in OC cells were measured according to the manufacturer’s instructions using Lactate Assay Kit (Solarbio, China), NAD⁺/NADH Assay Kit (Beyotime, China), and ATP Assay Kit (Elabscience, China), respectively. Briefly, cells were lysed and centrifuged to collect supernatants for analysis. For lactate measurement, samples were processed and read at 570 nm. NAD⁺/NADH levels were assessed after incubation, color development, and absorbance measurement at 450 nm. ATP levels were measured after cell lysis, incubation, and absorbance at 340 nm.

### LDH activity assay

Intracellular lactate dehydrogenase (LDH) activity was measured using a colorimetric LDH Activity Assay Kit (Elabscience, E-BC-K046-S) according to the manufacturer’s instructions. Briefly, cells were harvested and lysed, and clarified lysates were collected. LDH activity was determined based on a pyruvate standard curve by measuring absorbance at 450 nm. LDH activity was normalized to total protein concentration and expressed as relative LDH activity.

### Extracellular acidification rate (ECAR) and oxygen consumption rate (OCR) analysis

The extracellular acidification rate (ECAR) and oxygen consumption rate (OCR) were measured using a Seahorse XF Extracellular Flux Analyzer (Agilent Technologies) according to the manufacturer’s instructions. Briefly, OC cells with the indicated genetic manipulations were seeded into Seahorse XF cell culture microplates at an optimized density and allowed to adhere overnight. Prior to the assay, cells were washed and incubated in Seahorse XF assay medium supplemented with glucose, glutamine, and sodium pyruvate, and equilibrated in a non-CO₂ incubator at 37 °C for 1 h. For ECAR measurements, a glycolysis stress test was performed by sequential injection of glucose, oligomycin, and 2-deoxy-D-glucose. Basal glycolysis, glycolytic capacity, and glycolytic reserve were calculated according to standard Seahorse protocols. For OCR measurements, a mitochondrial stress test was conducted by sequential injection of oligomycin, carbonyl cyanide-p-trifluoromethoxyphenylhydrazone, and a mixture of rotenone and antimycin A. Basal respiration, maximal respiratory capacity, and spare respiratory capacity were determined. ECAR and OCR values were normalized to total protein content in each well. Data were analyzed using Seahorse Wave software and are presented as mean ± SD from at least three independent experiments.

### Immunofluorescence staining

The cells were rinsed with PBS, fixed in 4% paraformaldehyde for 20 min, and then permeabilized with 0.1% Triton X-100 in PBS for 10 min. Following a 30 min incubation with 5% BSA in PBS to block non-specific binding, the cells were incubated overnight at 4 °C with primary antibodies. Secondary antibodies were incubated for 1 h at room temperature, followed by DAPI staining of the nuclei for 5 min, with both steps carried out in the dark. Finally, coverslips were mounted, and images were captured using a fluorescence microscope.

### Statistical analysis

Statistical analyses and visualization were performed using R (version 4.1.3; https://www.r-project.org). To compare independent samples between two groups, the Student’s t-test was applied. For analyses involving more than two groups, one-way ANOVA was utilized. Survival outcomes were assessed via the log-rank test. Experimental results are expressed as the mean ± standard deviation (S.D.), derived from at least three independent experiments. A P-value below 0.05 was considered statistically significant (*p* < 0.05, *p < 0.01, **p < 0.001, ***p < 0.0001).

## Supplementary information

Supplementary table

Supplementary FigureS1

western blots

## Data Availability

The datasets generated during the current study are available in the TCGA (https://www.cancer.gov/tcga) and GTEx (https://www.gtexportal.org/home/) databases.
