# Supplementary material for: FGFR1 suppresses ovarian cancer progression by modulating SIRT3-dependent lactylation and metabolic reprogramming
Source: Cell Death Discov. 2026 Apr 7;12:244. doi: 10.1038/s41420-026-03054-6 (PMC13187239; doi:10.1038/s41420-026-03054-6)
Supplement: Supplementary file 1 — Supplementary table [file 41420_2026_3054_MOESM1_ESM.docx]

**Table 1. The sequences of shRNA and overexpression lentiviral constructs used in this study**

| **Name** | | **Target Sequences** |
| --- | --- | --- |
| **FGFR1-shRNA**  **FGFR1-OE** | ACCGGACGTGGAGTTCATGTGTAAGGTTCAAGAGACCTTACACATGAACTCCACGTTTTTTT  ATGTGGAGCTGGAAGTGCCTCCTCTTCTGGGCTGTGCTGGTCACAGCCACACTCTGCACCGCTAGGCCGTCCCCGACCTTGCCTGAACAAGCCCAGCCCTGGGGAGCCCCTGTGGAAGTGGAGTCCTTCCTGGTCCACCCCGGTGACCTGCTGCAGCTTCGCTGTCGGCTGCGGGACGATGTGCAGAGCATCAACTGGCTGCGGGACGGGGTGCAGCTGGCGGAAAGCAACCGCACCCGCATCACAGGGGAGGAGGTGGAGGTGCAGGACTCCGTGCCCGCAGACTCCGGCCTCTATGCTTGCGTAACCAGCAGCCCCTCGGGCAGTGACACCACCTACTTCTCCGTCAATGTTTCAGATGCTCTCCCCTCCTCGGAGGATGATGATGATGATGATGACTCCTCTTCAGAGGAGAAAGAAACAGATAACACCAAACCAAACCGTATGCCCGTAGCTCCATATTGGACATCCCCAGAAAAGATGGAAAAGAAATTGCATGCAGTGCCGGCTGCCAAGACAGTGAAGTTCAAATGCCCTTCCAGTGGGACCCCAAACCCCACACTGCGCTGGTTGAAAAATGGCAAAGAATTCAAACCTGACCACAGAATTGGAGGCTACAAGGTCCGTTATGCCACCTGGAGCATCATAATGGACTCTGTGGTGCCCTCTGACAAGGGCAACTACACCTGCATTGTGGAGAATGAGTACGGCAGCATCAACCACACATACCAGCTGGATGTCGTGGAGCGGTCCCCTCACCGGCCCATCCTGCAAGCAGGGTTGCCCGCCAACAAAACAGTGGCCCTGGGTAGCAACGTGGAGTTCATGTGTAAGGTGTACAGTGACCCGCAGCCGCACATCCAGTGGCTAAAGCACATCGAGGTGAATGGGAGCAAGATTGGCCCAGACAACCTGCCTTATGTCCAGATCTTGAAGACTGCTGGAGTTAATACCACCGACAAAGAGATGGAGGTGCTTCACTTAAGAAATGTCTCCTTTGAGGACGCAGGGGAGTATACGTGCTTGGCGGGTAACTCTATCGGACTCTCCCATCACTCTGCATGGTTGACCGTTCTGGAAGCCCTGGAAGAGAGGCCGGCAGTGATGACCTCGCCCCTGTACCTGGAGATCATCATCTATTGCACAGGGGCCTTCCTCATCTCCTGCATGGTGGGGTCGGTCATCGTCTACAAGATGAAGAGTGGTACCAAGAAGAGTGACTTCCACAGCCAGATGGCTGTGCACAAGCTGGCCAAGAGCATCCCTCTGCGCAGACAGGTAACAGTGTCTGCTGACTCCAGTGCATCCATGAACTCTGGGGTTCTTCTGGTTCGGCCATCACGGCTCTCCTCCAGTGGGACTCCCATGCTAGCAGGGGTCTCTGAGTATGAGCTTCCCGAAGACCCTCGCTGGGAGCTGCCTCGGGACAGACTGGTCTTAGGCAAACCCCTGGGAGAGGGCTGCTTTGGGCAGGTGGTGTTGGCAGAGGCTATCGGGCTGGACAAGGACAAACCCAACCGTGTGACCAAAGTGGCTGTGAAGATGTTGAAGTCGGACGCAACAGAGAAAGACTTGTCAGACCTGATCTCAGAAATGGAGATGATGAAGATGATCGGGAAGCATAAGAATATCATCAACCTGCTGGGGGCCTGCACGCAGGATGGTCCCTTGTATGTCATCGTGGAGTATGCCTCCAAGGGCAACCTGCGGGAGTACCTGCAGGCCCGGAGGCCCCCAGGGCTGGAATACTGCTACAACCCCAGCCACAACCCAGAGGAGCAGCTCTCCTCCAAGGACCTGGTGTCCTGCGCCTACCAGGTGGCCCGAGGCATGGAGTATCTGGCCTCCAAGAAGTGCATACACCGAGACCTGGCAGCCAGGAATGTCCTGGTGACAGAGGACAATGTGATGAAGATAGCAGACTTTGGCCTCGCACGGGACATTCACCACATCGACTACTATAAAAAGACAACCAACGGCCGACTGCCTGTGAAGTGGATGGCACCCGAGGCATTATTTGACCGGATCTACACCCACCAGAGTGATGTGTGGTCTTTCGGGGTGCTCCTGTGGGAGATCTTCACTCTGGGCGGCTCCCCATACCCCGGTGTGCCTGTGGAGGAACTTTTCAAGCTGCTGAAGGAGGGTCACCGCATGGACAAGCCCAGTAACTGCACCAACGAGCTGTACATGATGATGCGGGACTGCTGGCATGCAGTGCCCTCACAGAGACCCACCTTCAAGCAGCTGGTGGAAGACCTGGACCGCATCGTGGCCTTGACCTCCAACCAGGAGTACCTGGACCTGTCCATGCCCCTGGACCAGTACTCCCCCAGCTTTCCCGACACCCGGAGCTCTACGTGCTCCTCAGGGGAGGATTCCGTCTTCTCTCATGAGCCGCTGCCCGAGGAGCCCTGCCTGCCCCGACACCCAGCCCAGCTTGCCAATGGCGGACTCAAACGCCGC | |
| **SIRT3-shRNA** | GTGGAAGAAGGTCCATATCTTTT | |
| **SIRT3-OE** | ATGGCGTTCTGGGGTTGGCGCGCCGCGGCAGCCCTCCGGCTGTGGGGCCGGGTAGTTGAACGGGTCGAGGCCGGGGGAGGCGTGGGGCCGTTTCAGGCCTGCGGCTGTCGGCTGGTGCTTGGCGGCAGGGACGATGTGAGTGCGGGGCTGAGAGGCAGCCATGGGGCCCGCGGTGAGCCCTTGGACCCGGCGCGCCCCTTGCAGAGGCCTCCCAGACCCGAGGTGCCCAGGGCATTCCGGAGGCAGCCGAGGGCAGCAGCTCCCAGTTTCTTCTTTTCGAGTATTAAAGGTGGAAGAAGGTCCATATCTTTTTCTGTGGGTGCTTCAAGTGTTGTTGGAAGTGGAGGCAGCAGTGACAAGGGGAAGCTTTCCCTGCAGGATGTAGCTGAGCTGATTCGGGCCAGAGCCTGCCAGAGGGTGGTGGTCATGGTGGGGGCCGGCATCAGCACACCCAGTGGCATTCCAGACTTCAGATCGCCGGGGAGTGGCCTGTACAGCAACCTCCAGCAGTACGATCTCCCGTACCCCGAGGCCATTTTTGAACTCCCATTCTTCTTTCACAACCCCAAGCCCTTTTTCACTTTGGCCAAGGAGCTGTACCCTGGAAACTACAAGCCCAACGTCACTCACTACTTTCTCCGGCTGCTTCATGACAAGGGGCTGCTTCTGCGGCTCTACACGCAGAACATCGATGGGCTTGAGAGAGTGTCGGGCATCCCTGCCTCAAAGCTGGTTGAAGCTCATGGAACCTTTGCCTCTGCCACCTGCACAGTCTGCCAAAGACCCTTCCCAGGGGAGGACATTCGGGCTGACGTGATGGCAGACAGGGTTCCCCGCTGCCCGGTCTGCACCGGCGTTGTGAAGCCCGACATTGTGTTCTTTGGGGAGCCGCTGCCCCAGAGGTTCTTGCTGCATGTGGTTGATTTCCCCATGGCAGATCTGCTGCTCATCCTTGGGACCTCCCTGGAGGTGGAGCCTTTTGCCAGCTTGACCGAGGCCGTGCGGAGCTCAGTTCCCCGACTGCTCATCAACCGGGACTTGGTGGGGCCCTTGGCTTGGCATCCTCGCAGCAGGGACGTGGCCCAGCTGGGGGACGTGGTTCACGGCGTGGAAAGCCTAGTGGAGCTTCTGGGCTGGACAGAAGAGATGCGGGACCTTGTGCAGCGGGAAACTGGGAAGCTTGATGGACCAGACAAATAG | |

**Table 2.** **Sequences of primers used in this study**

| **Name** | **Sequences of Primer** |
| --- | --- |
| GAPDH-F  GAPDH-R  FGFR1-F  FGFR1-R  HDAC2-F  HDAC2-R  HK2-F  HK2-R  PFKM-F  PFKM-R  SIRT3-F  SIRT3-R | ATCATCAGCAATGCCTCC CATCACGCCACAGTTTCC  TCAAATGCCCTTCCAGTG CATAACGGACCTTGTAGCC  ATATTGTGCTTGCCATCC  CCTCAAGTCTCCTGTGCC  AGGTCCTGATGCGGTTGG  TCGCCTTTGTTCTCCTTGAT  ACCCGTGGTTCTCGTCTC  AAAGGCTGATGGCGTCCC  AGGGTGGTGGTCATGGTG  TTGTGAAAGAAGAATGGGAGTT |

**Table 3. Antibodies utilized in the study**

| **Antibody** | **Company** | **Cat. Number** | **Dilution** |
| --- | --- | --- | --- |
| FGFR1  GAPDH  PanKla  H3K18la  LDHA  LDHB  CCND1  CCNE1  SIRT3 | CST  CST  ABclonal  PTM Bio  proteintech  proteintech  CST  CST  proteintech | 9740S  2118S  A23004  PTM-1427RM  21799-1-AP  66425-1-Ig  55506  20808  10099-1-AP | 1:1000  1:1000  1:500  1:500  1:5000  1:20000  1:1000  1:1000  1:2000 |
