# Supplementary material for: FGFR1 suppresses ovarian cancer progression by modulating SIRT3-dependent lactylation and metabolic reprogramming
Source: Cell Death Discov. 2026 Apr 7;12:244. doi: 10.1038/s41420-026-03054-6 (PMC13187239; doi:10.1038/s41420-026-03054-6)
Supplement: Supplementary file 2 — Supplementary FigureS1 [file 41420_2026_3054_MOESM2_ESM.docx]

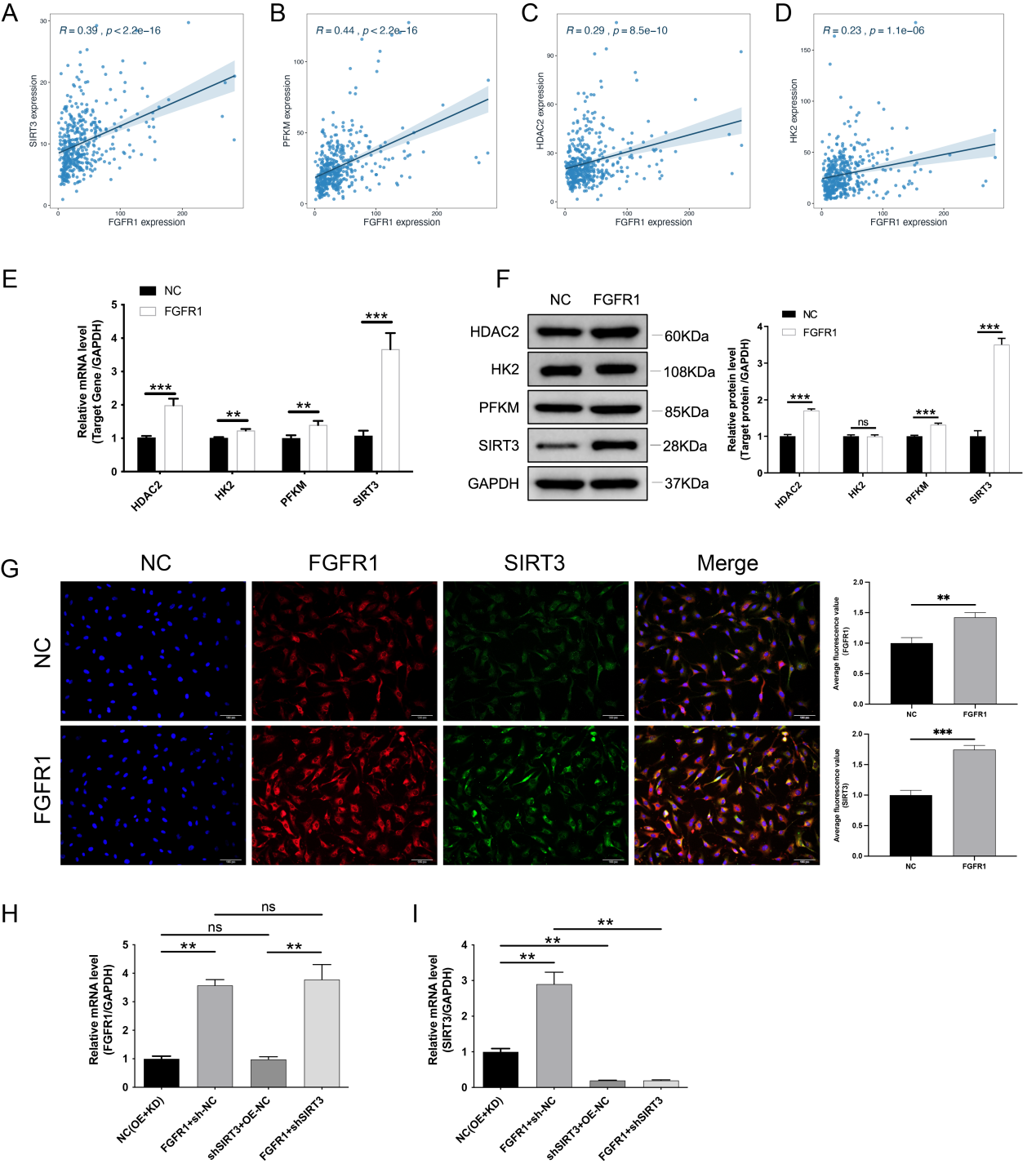


**Figure S1.** FGFR1 Positively Correlates With Metabolic Regulators and Upregulates SIRT3 in OC Cells. (A–D) Correlation analysis of TCGA-OV cohort showing that FGFR1 expression is positively associated with SIRT3 (A), PFKM (B), HDAC2 (C), and HK2 (D). (E) qRT-PCR analysis demonstrating that FGFR1 overexpression significantly increases the mRNA levels of HDAC2, HK2, PFKM, and SIRT3 in OVCAR-3 cells. (F) Western blot results confirming elevated protein levels of HDAC2, HK2, PFKM, and SIRT3 upon FGFR1 overexpression; quantification shown on the right. (G) Immunofluorescence staining showing increased FGFR1 and SIRT3 fluorescence intensity in FGFR1-overexpressing cells compared with NC; quantification of fluorescence intensity is shown on the right. (H–I) qRT-PCR analysis showing that SIRT3 knockdown attenuates the FGFR1-induced upregulation of FGFR1 (H) and SIRT3 (I).
