# Supplementary material for: FGFR1 suppresses ovarian cancer progression by modulating SIRT3-dependent lactylation and metabolic reprogramming
Source: Cell Death Discov. 2026 Apr 7;12:244. doi: 10.1038/s41420-026-03054-6 (PMC13187239; doi:10.1038/s41420-026-03054-6)
Supplement: Supplementary file 3 — western blots [file 41420_2026_3054_MOESM3_ESM.docx]

FGFR1 overexpression was verified（OVCAR-3）:

FGFR1 GAPDH


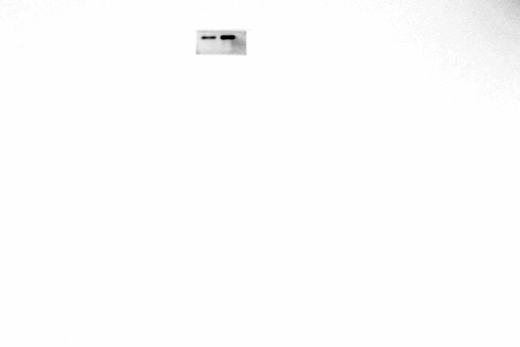

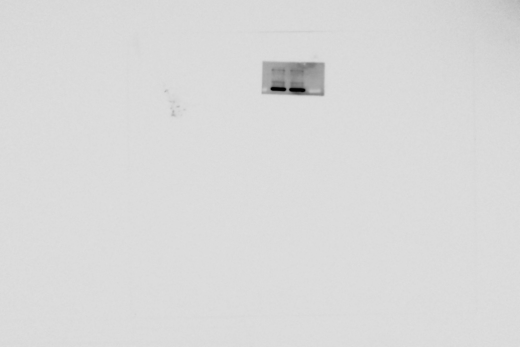


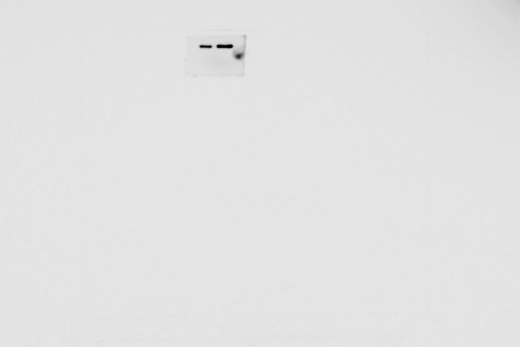

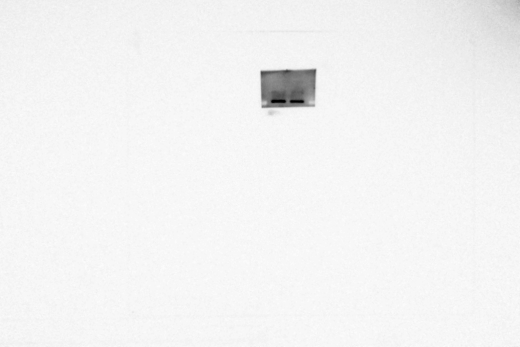


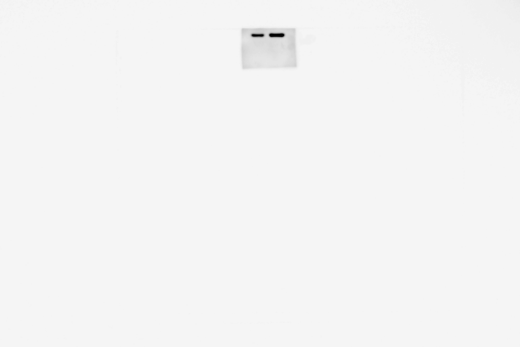

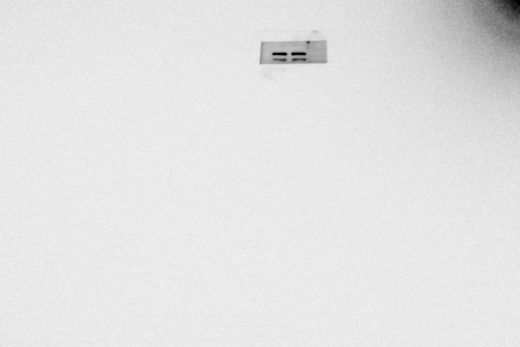


FGFR1 overexpression was verified（SKOV-3）:

FGFR1 GAPDH


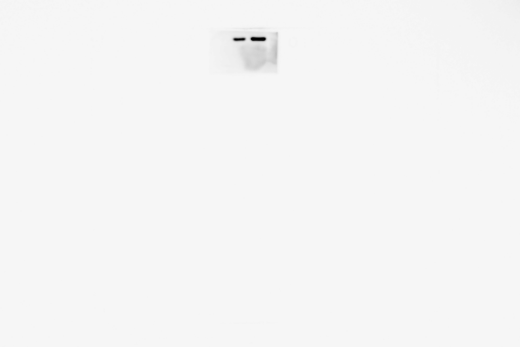

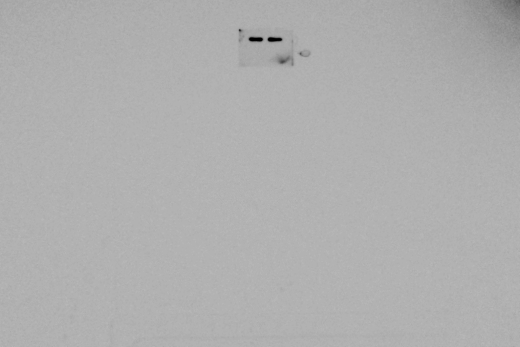


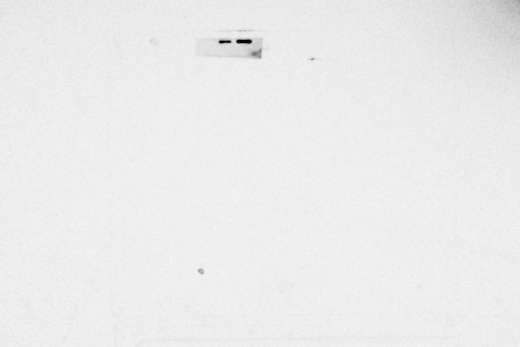

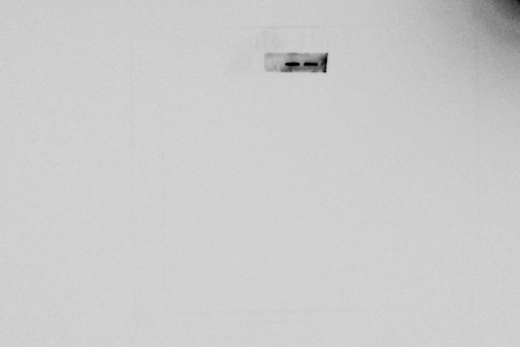


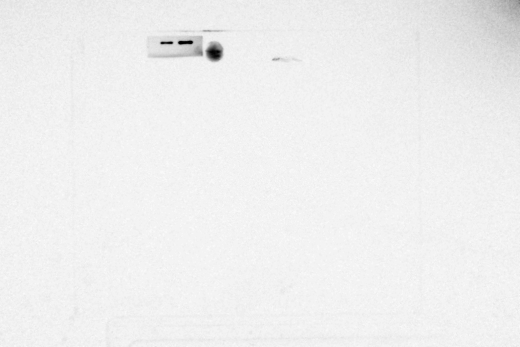

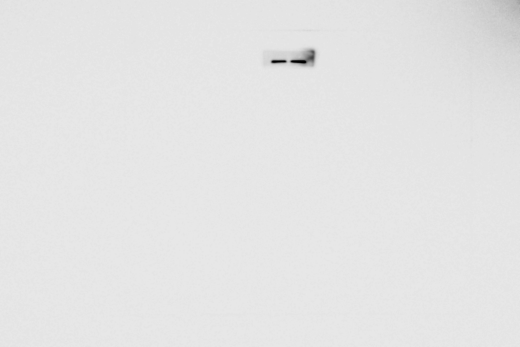


FGFR1 knockdown validation（OVCAR-3）:

FGFR1 GAPDH


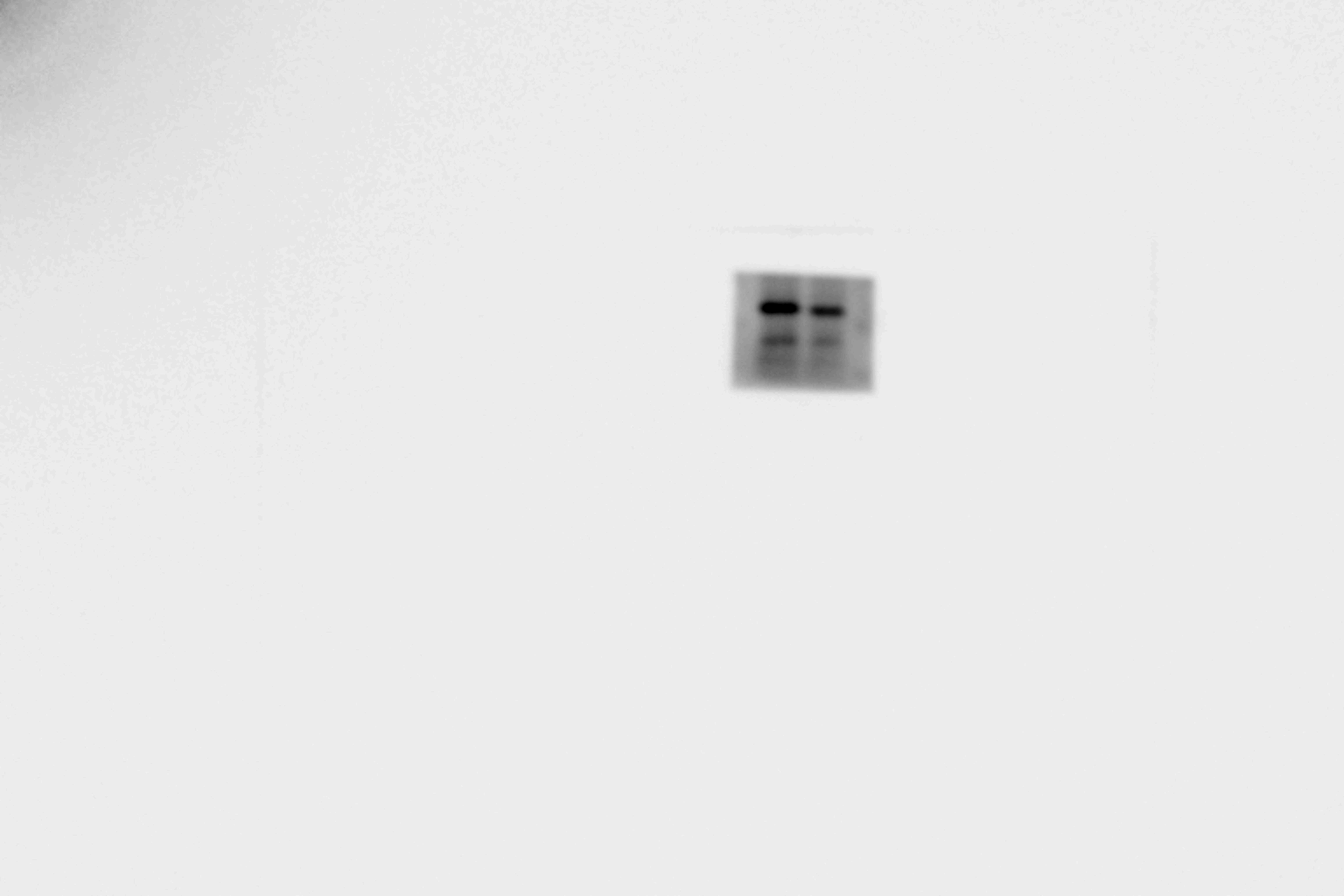

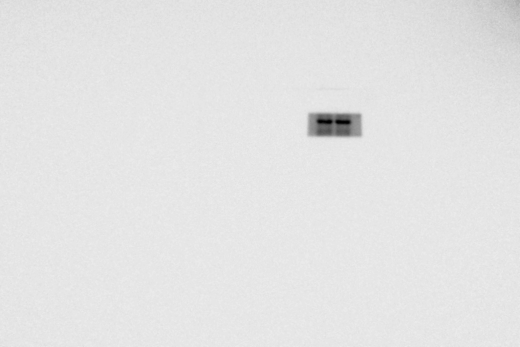


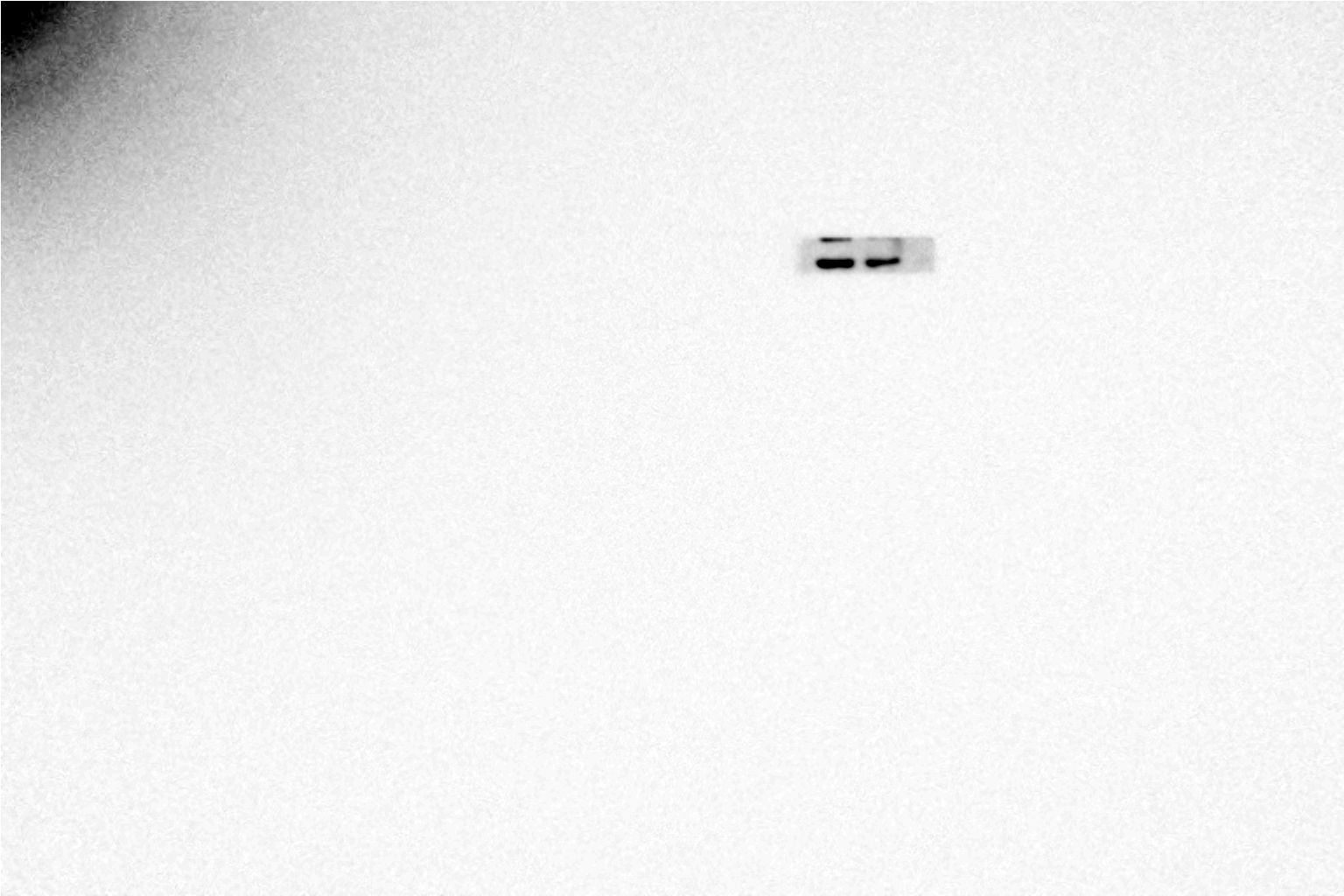

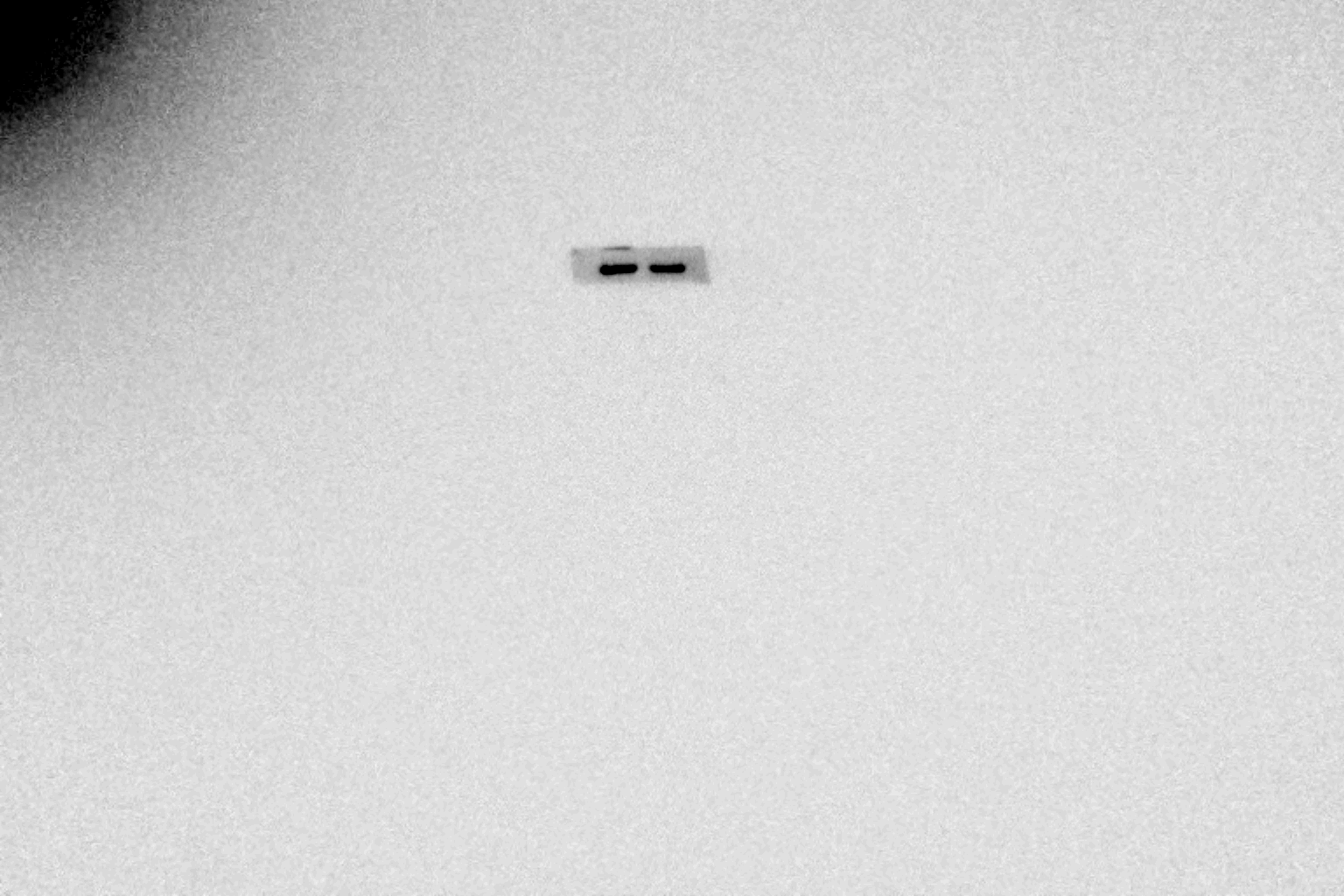


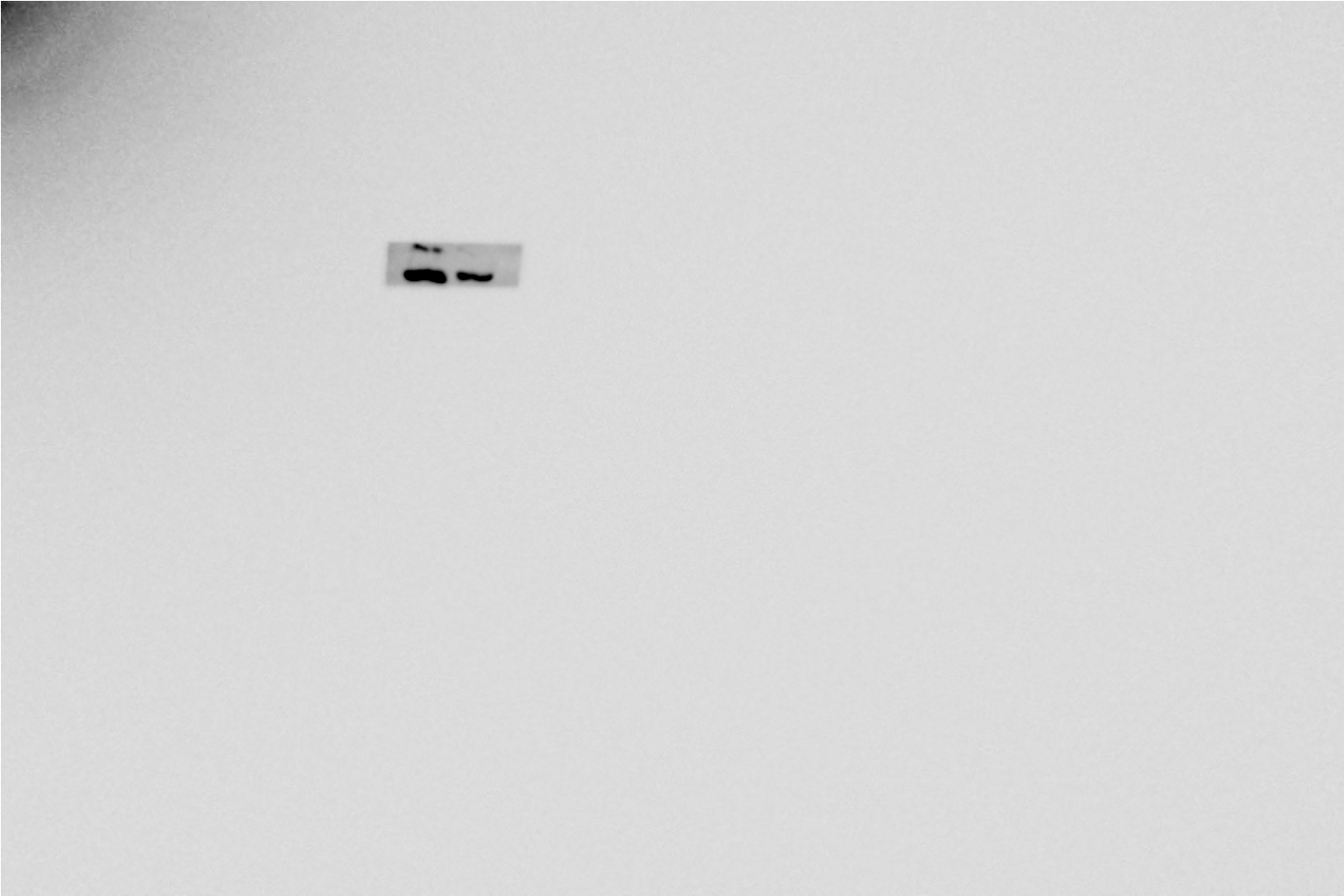

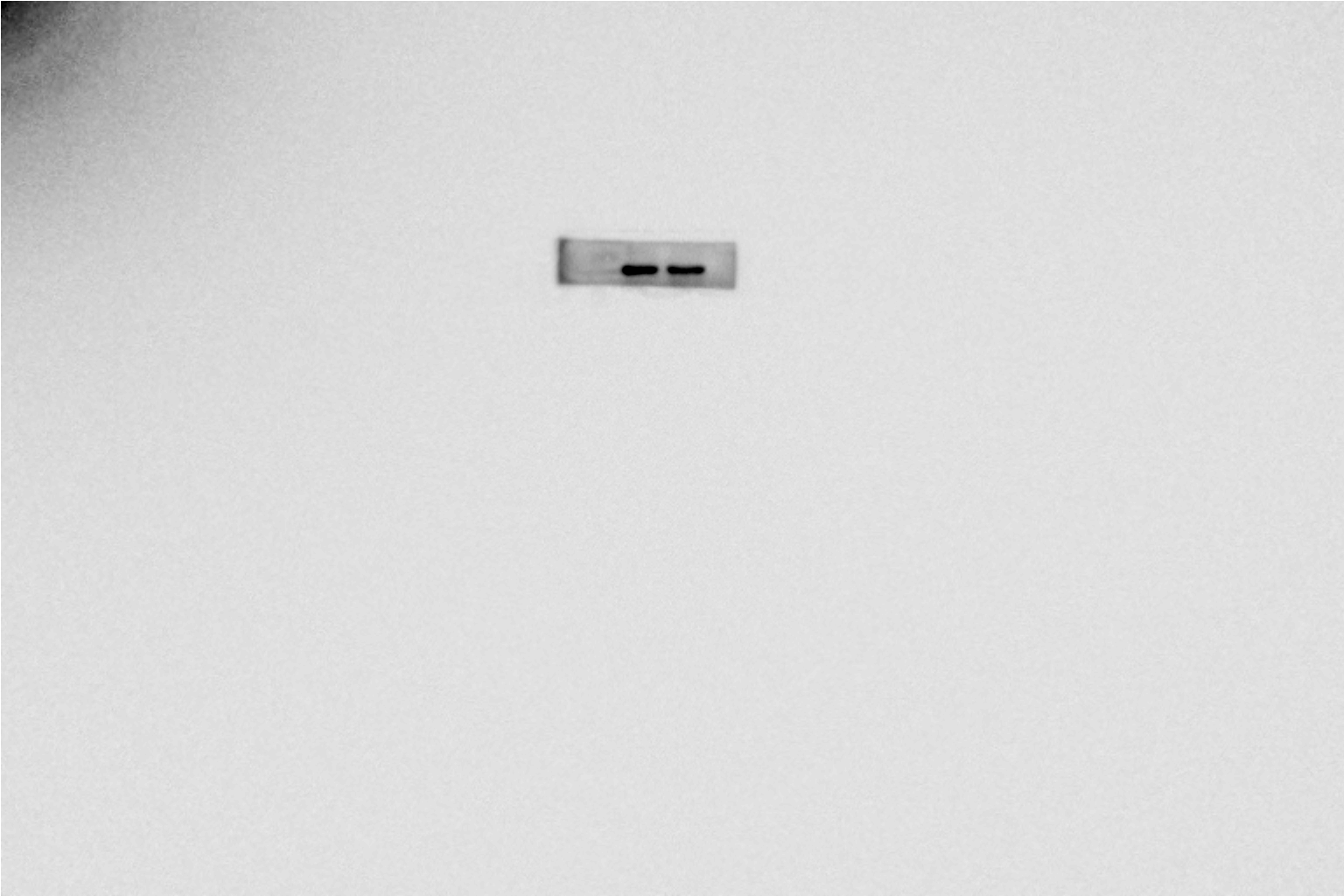


FGFR1 knockdown validation（SKOV-3）:

FGFR1 GAPDH


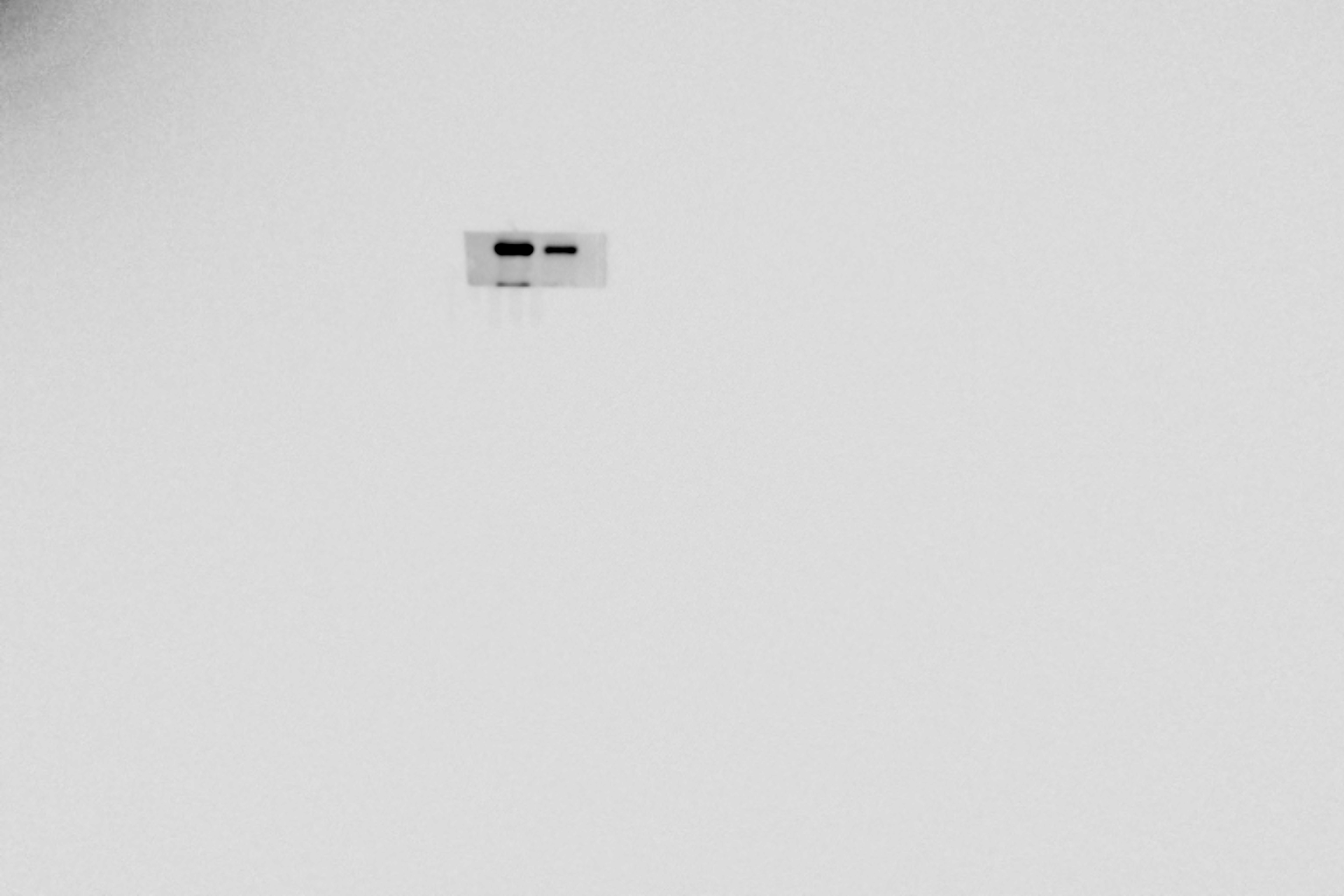

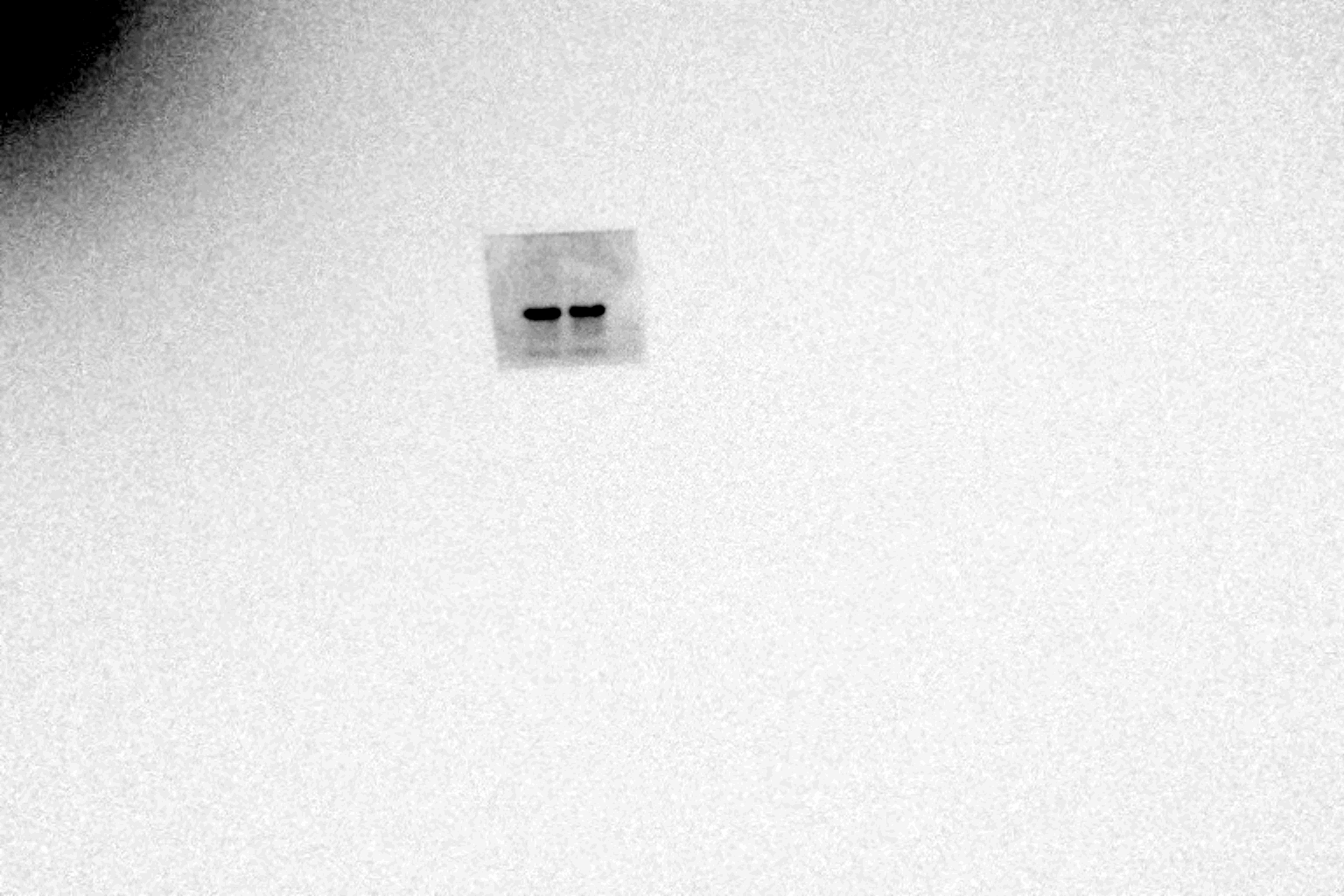


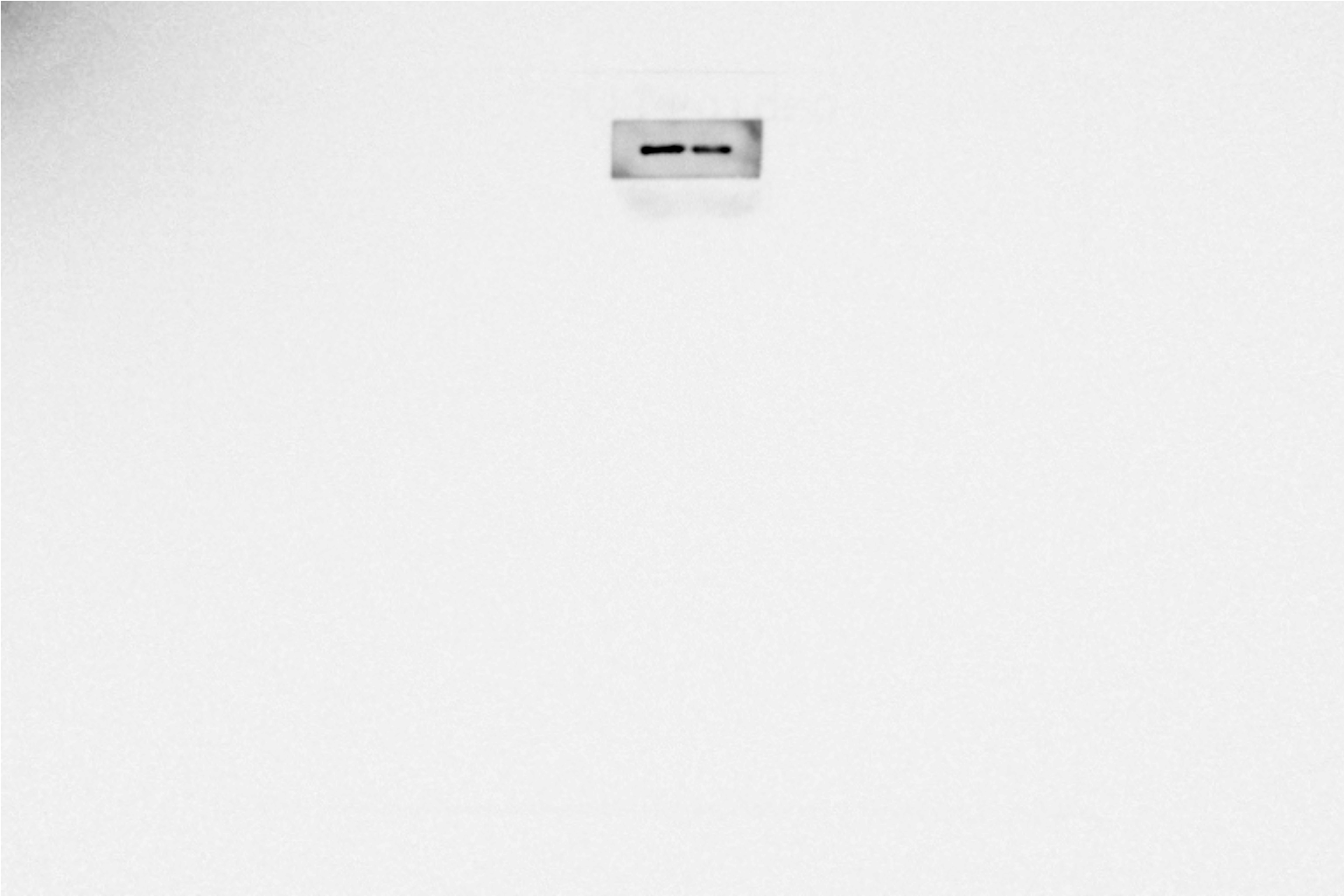

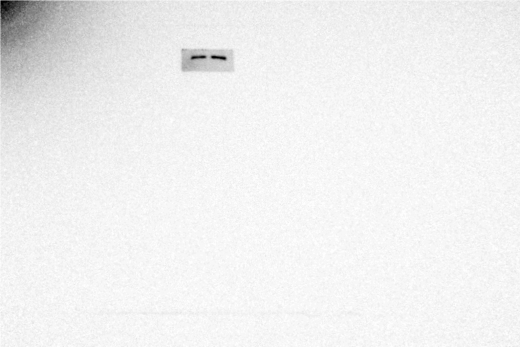


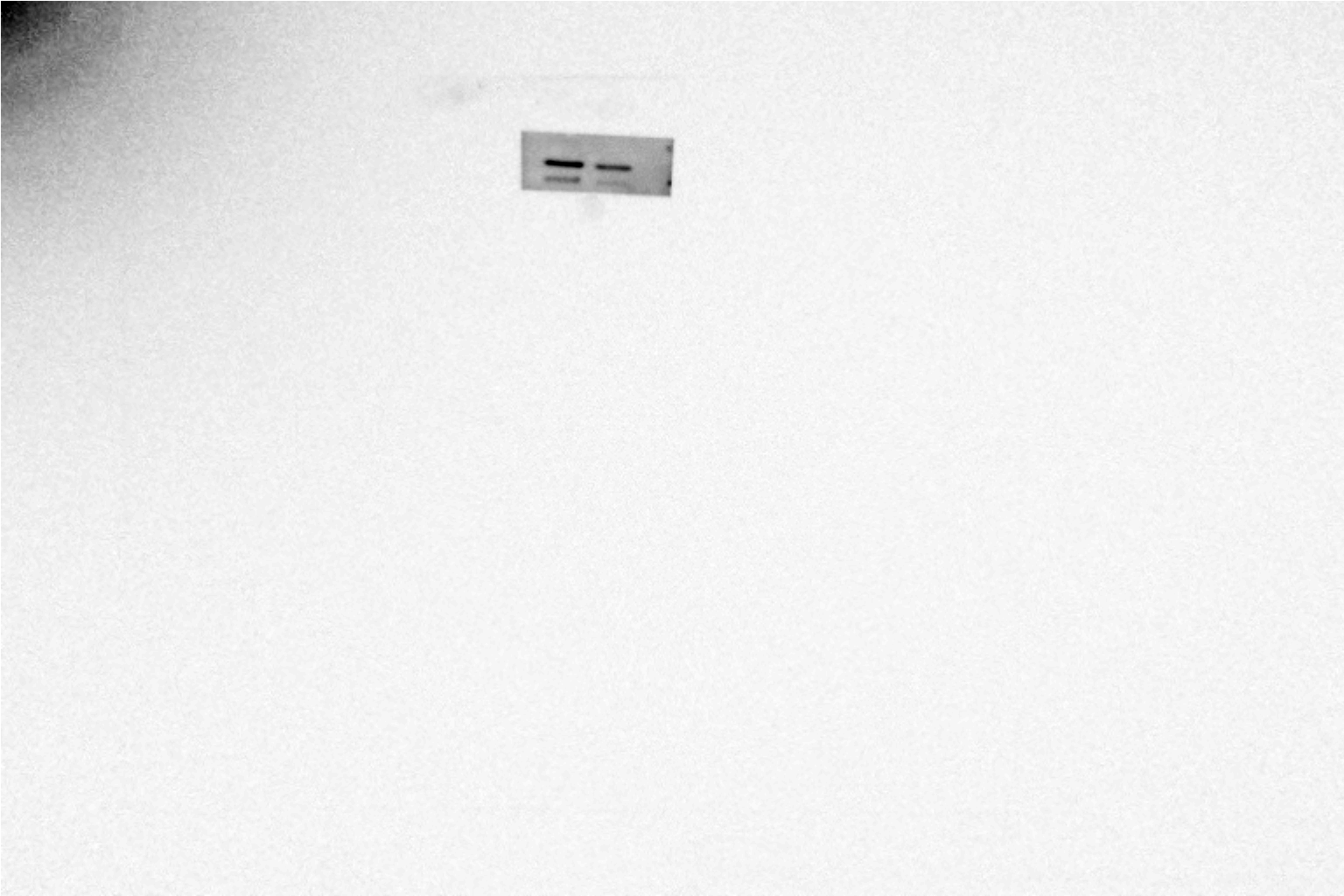

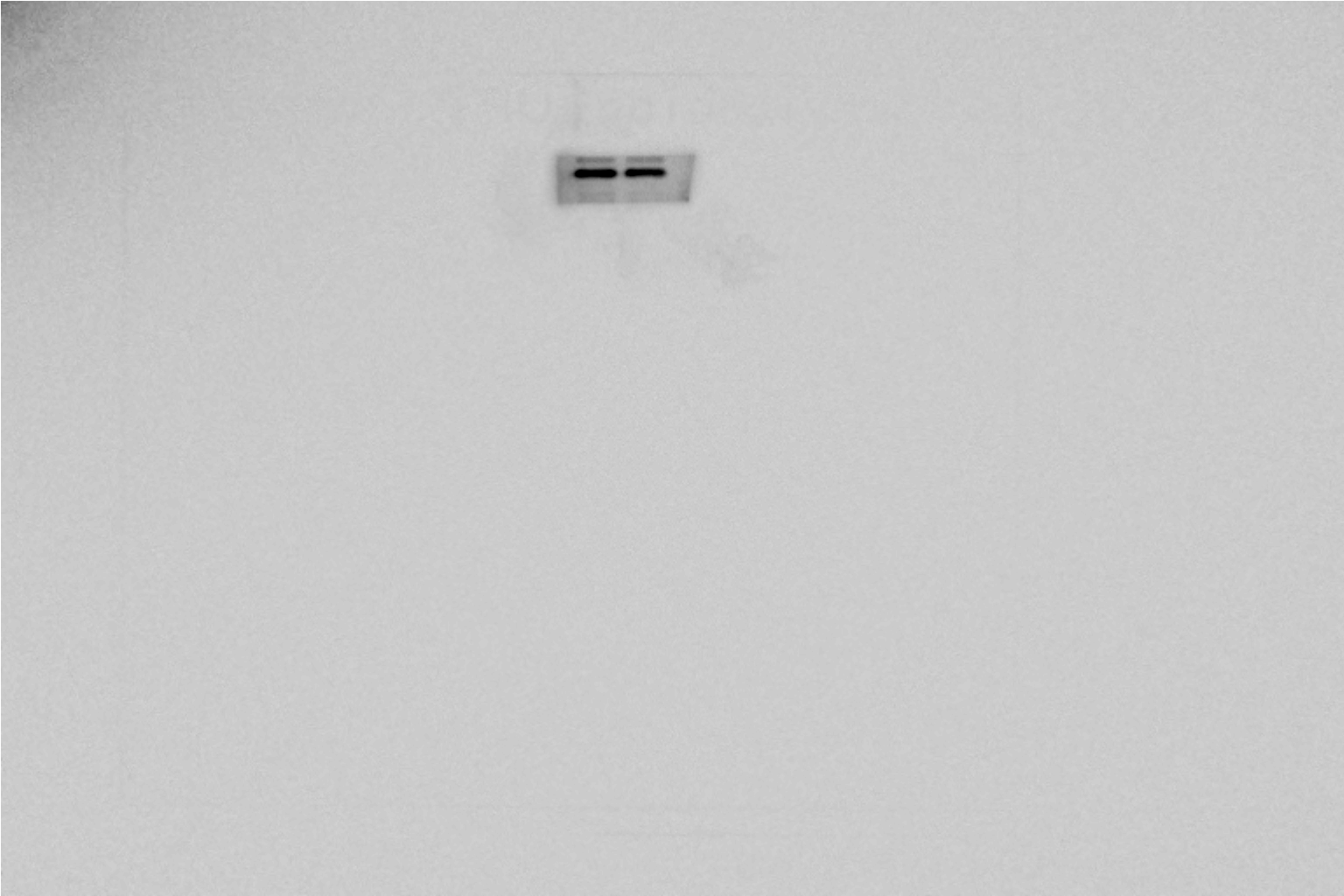


LDHA and LDHB（OVCAR-3）:

LDHA LDHB GAPDH


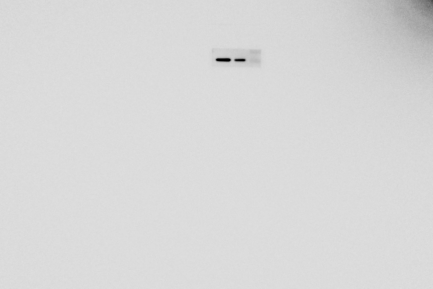

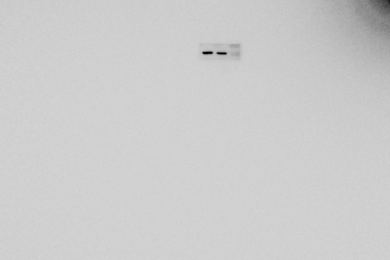

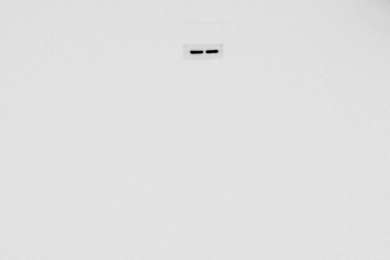


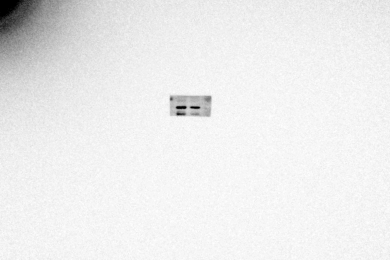

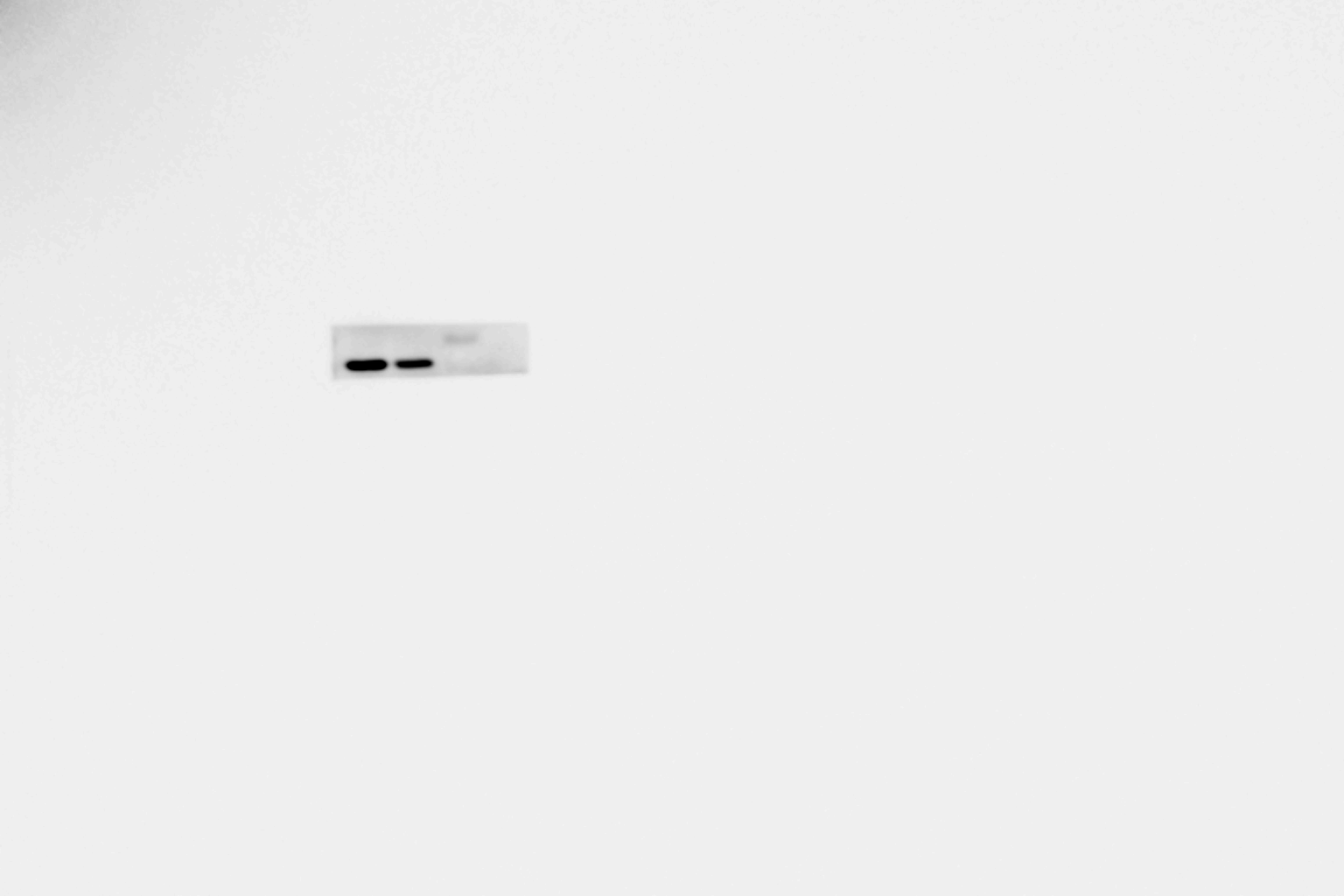

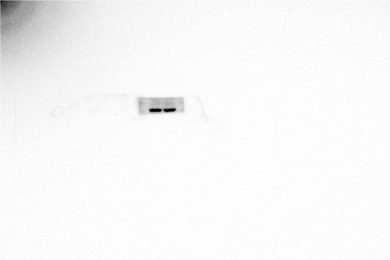


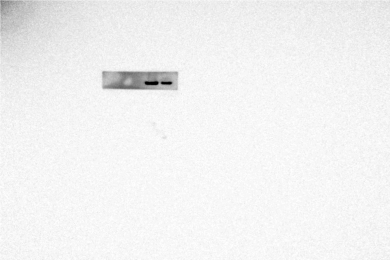

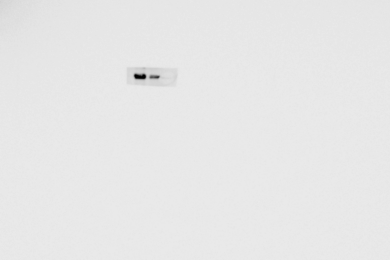

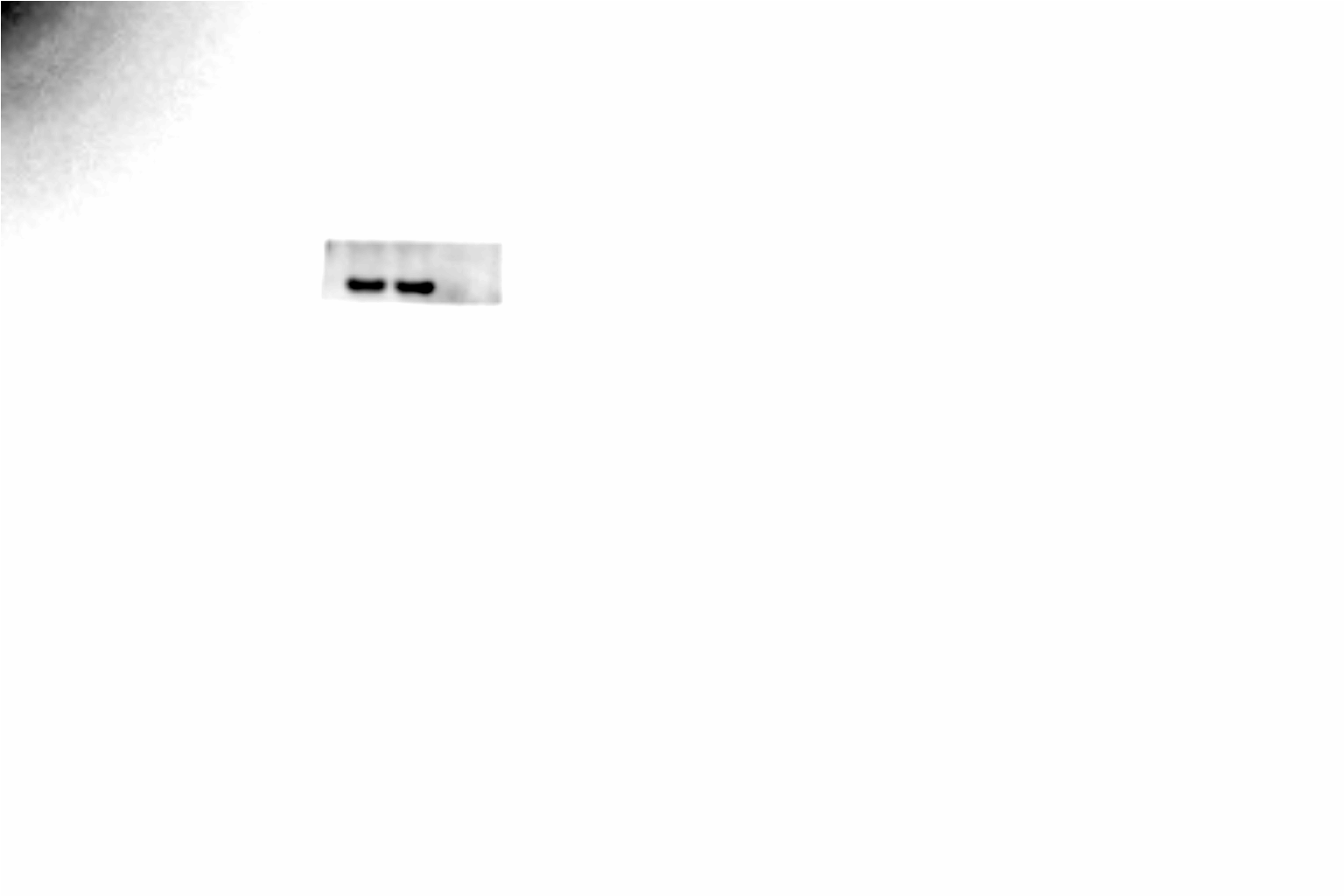


LDHA and LDHB（SKOV-3）：

LDHA LDHB GAPDH


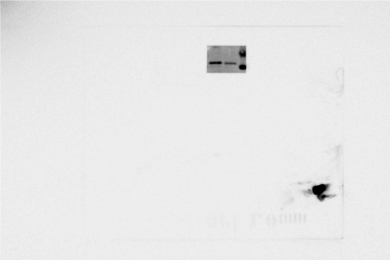

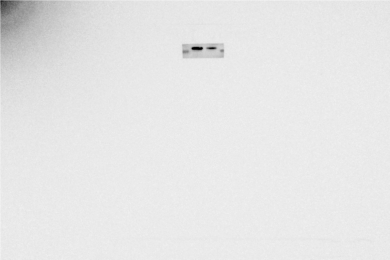

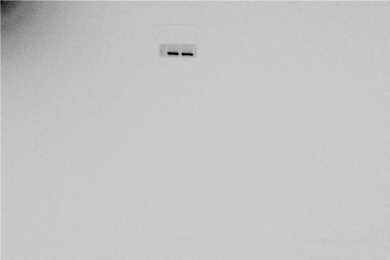


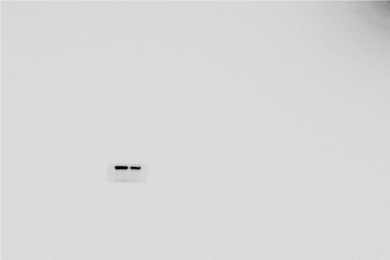

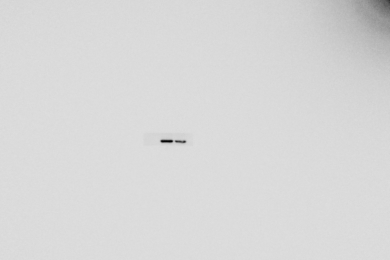

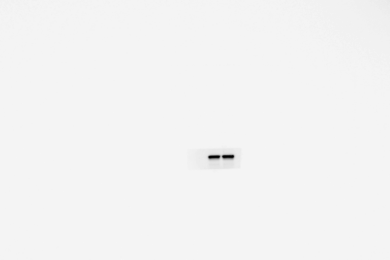


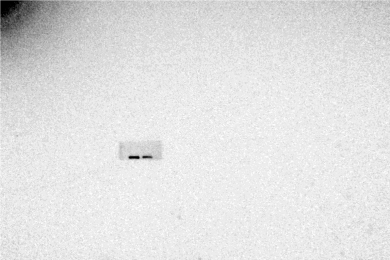

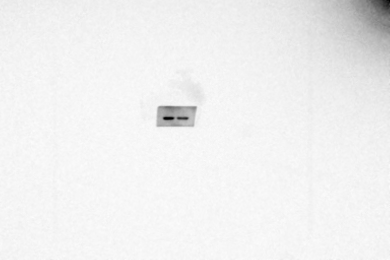

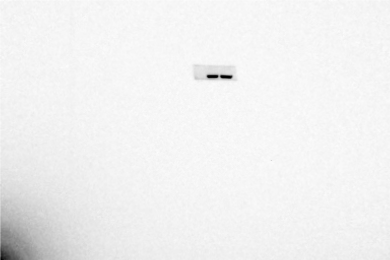


Lactate co-cultivation was used to detect lactation levels（OVCAR-3）：

H3 H3K18la Pankla


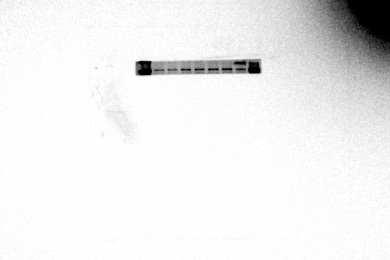

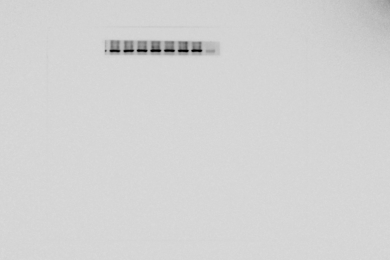

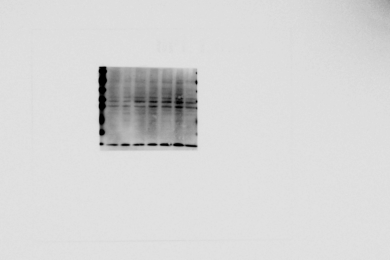


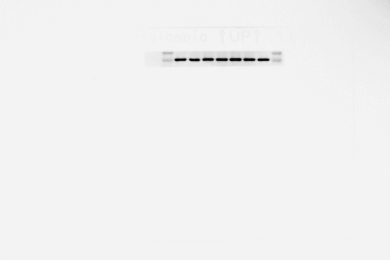

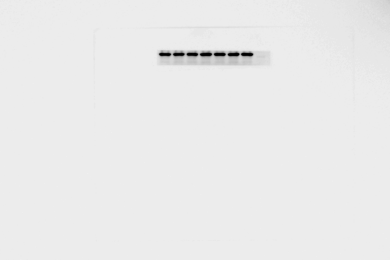

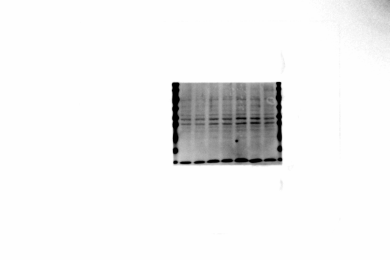


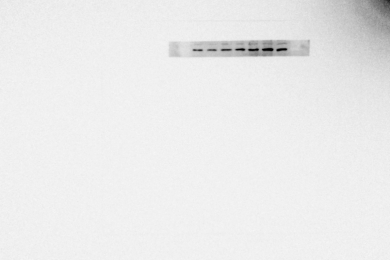

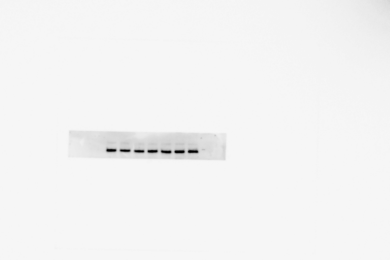

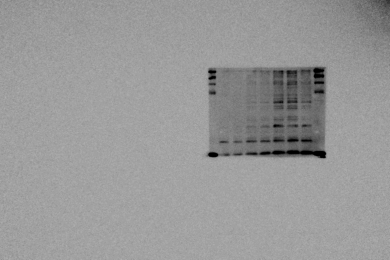


Lactate co-culture was used to detect cyclin levels（OVCAR-3）：

CCND1 CCNE1 GADPH


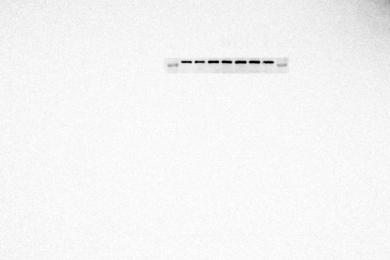

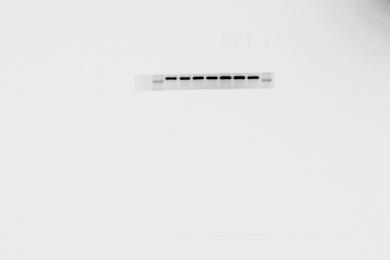

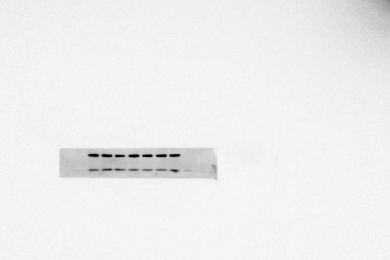


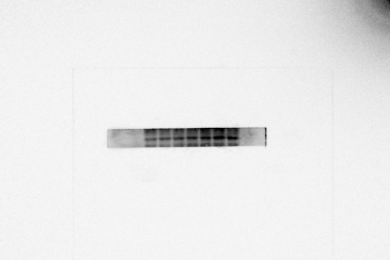

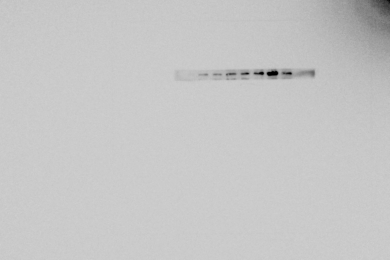

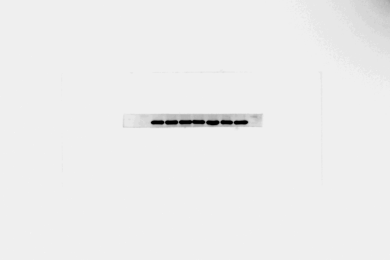


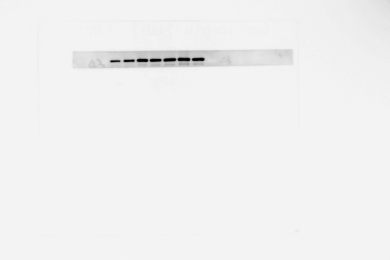

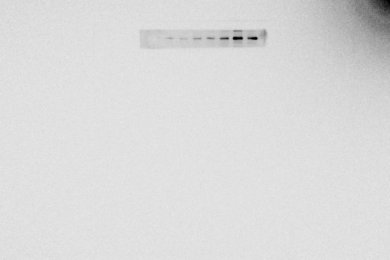

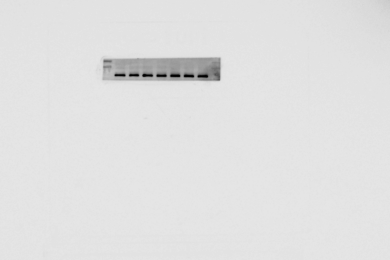


Lactate co-cultivation was used to detect lactation levels（SKOV-3）：

H3 H3K18la Pankla


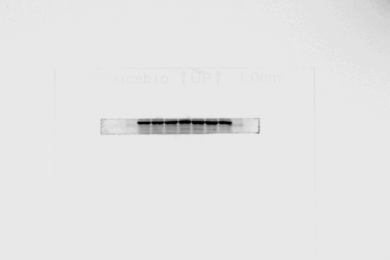

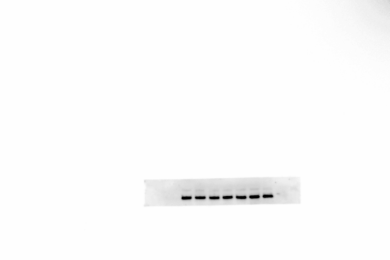

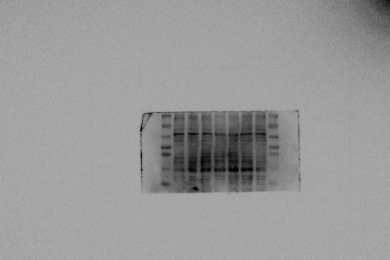


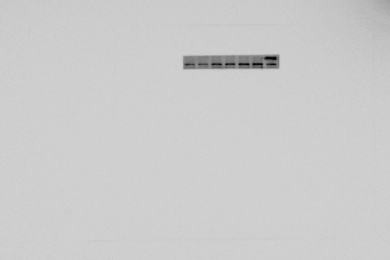

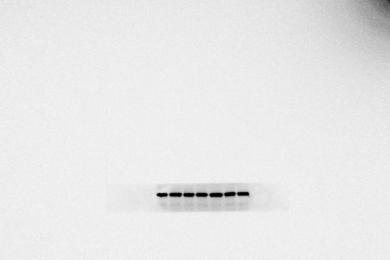

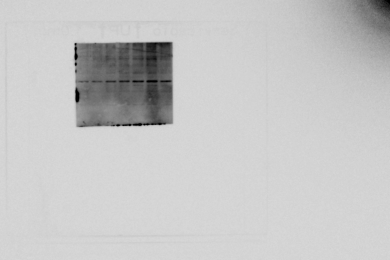


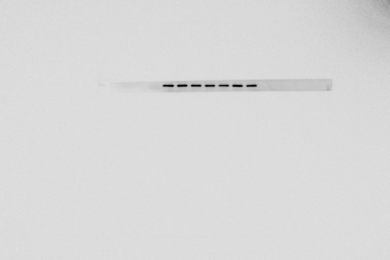

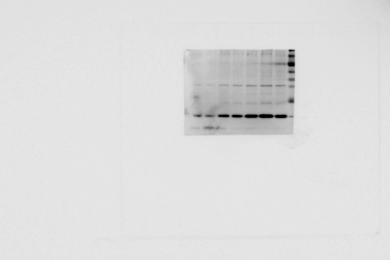

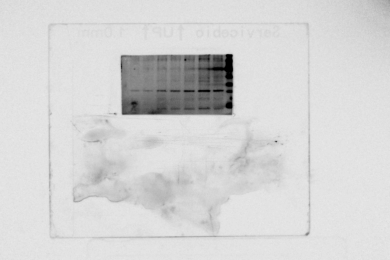


Lactate co-culture was used to detect cyclin levels（SKOV-3）：

CCND1 CCNE1 GADPH


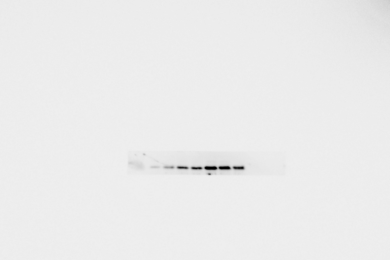

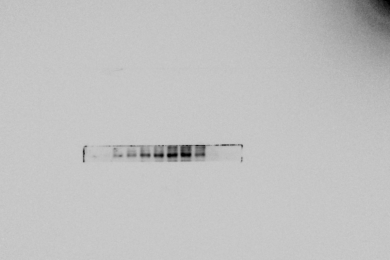

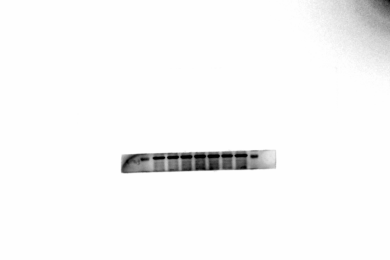


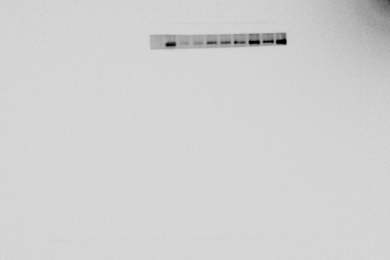

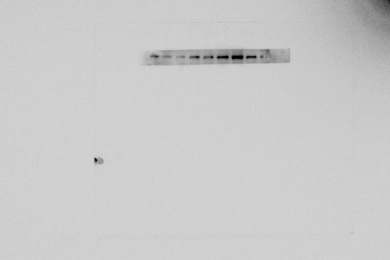

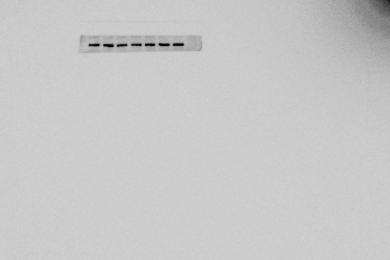


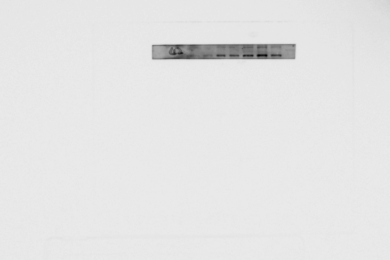

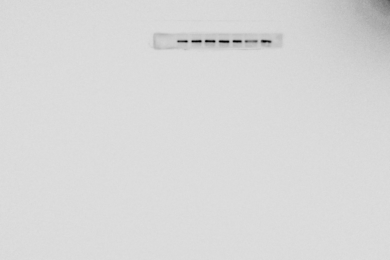

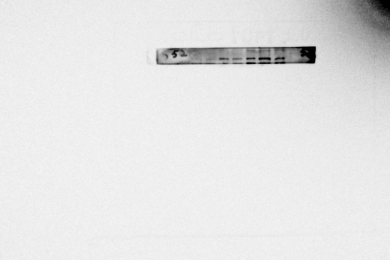


Lactination level（OVCAR-3）：

H3 H3K18la Pankla


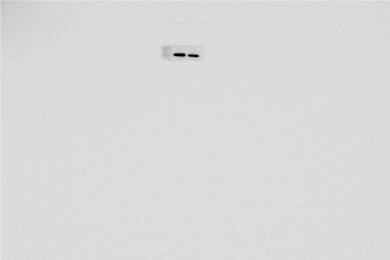

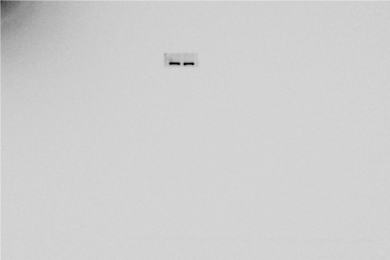

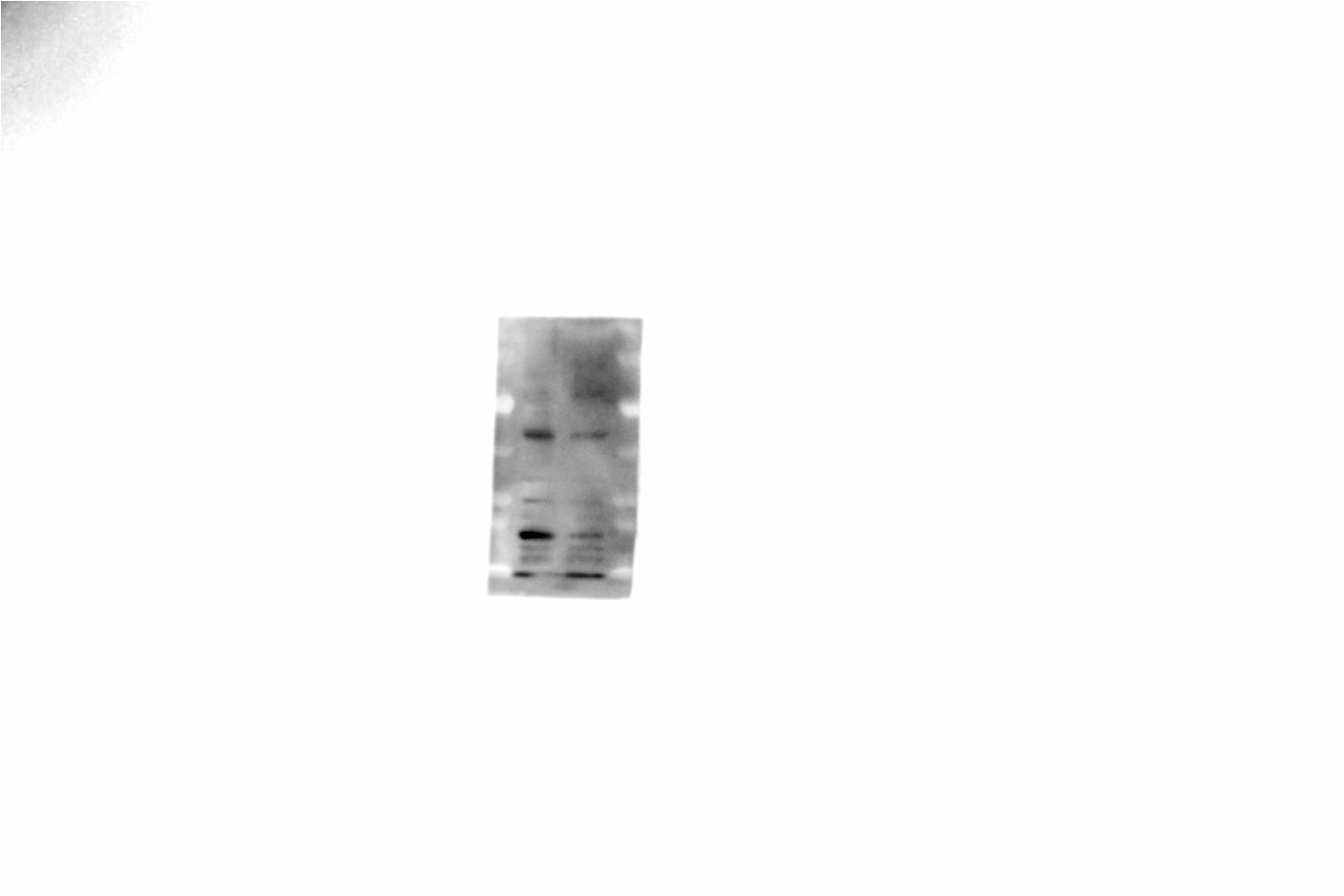


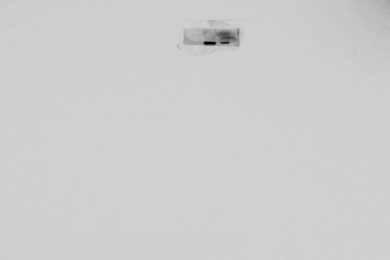

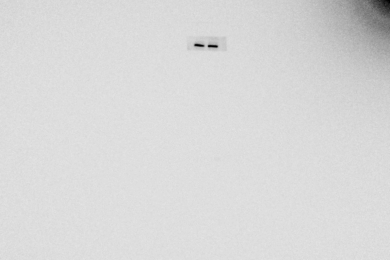

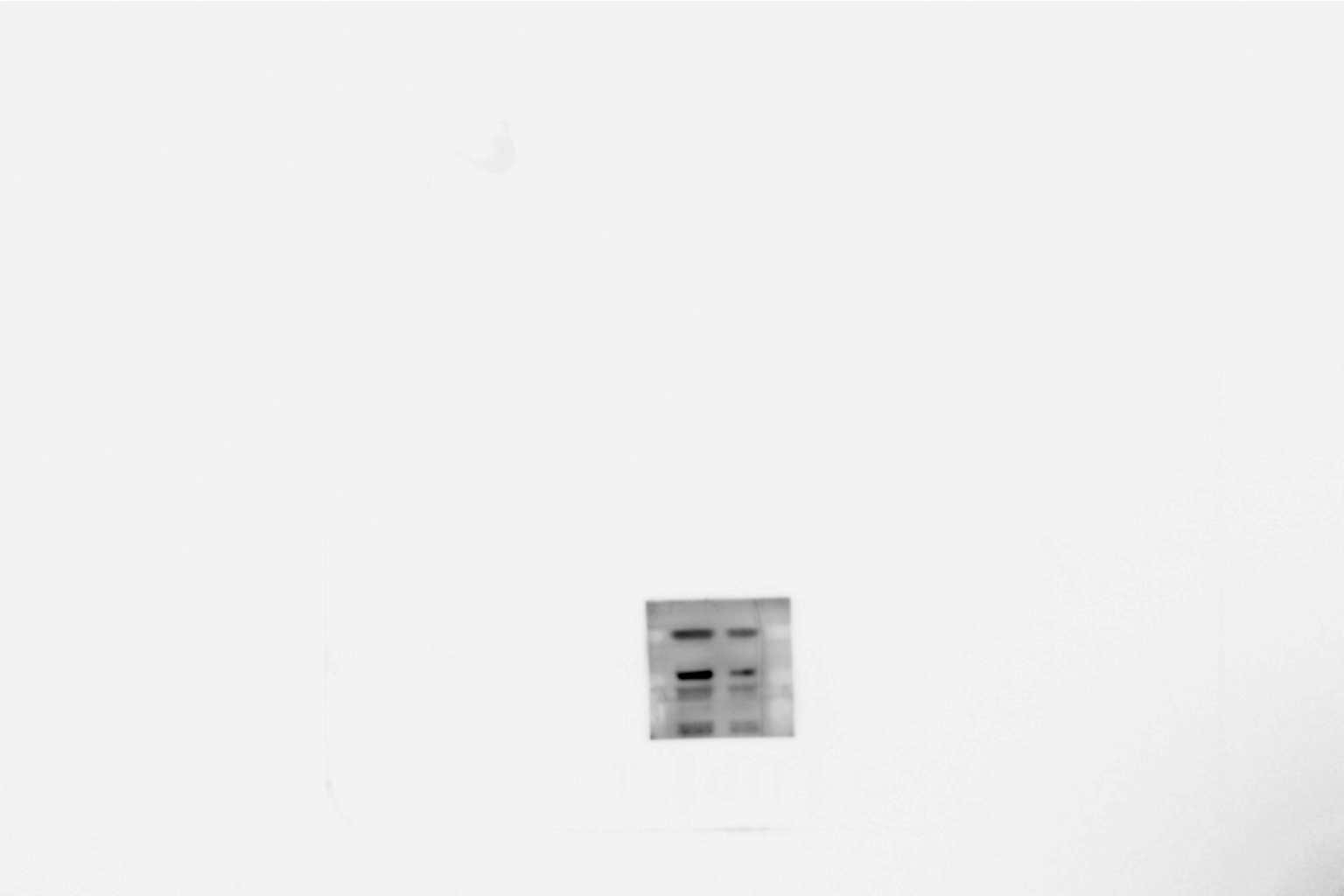


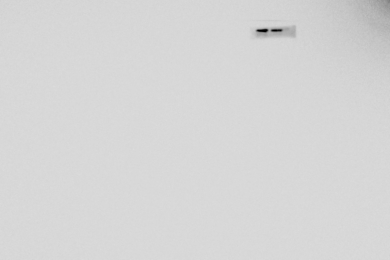

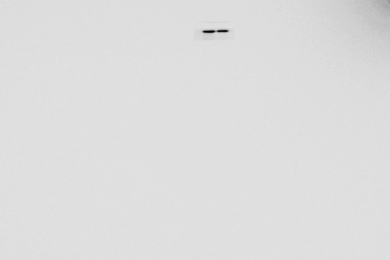

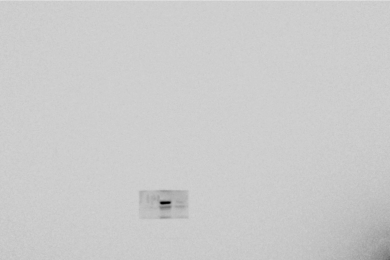


Lactination level（SKOV-3）：

H3 H3K18la Pankla


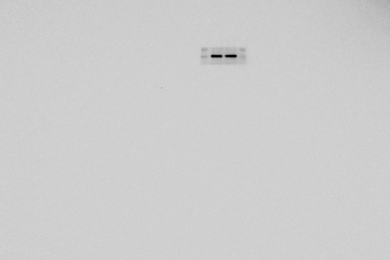

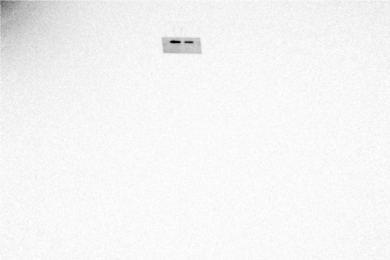

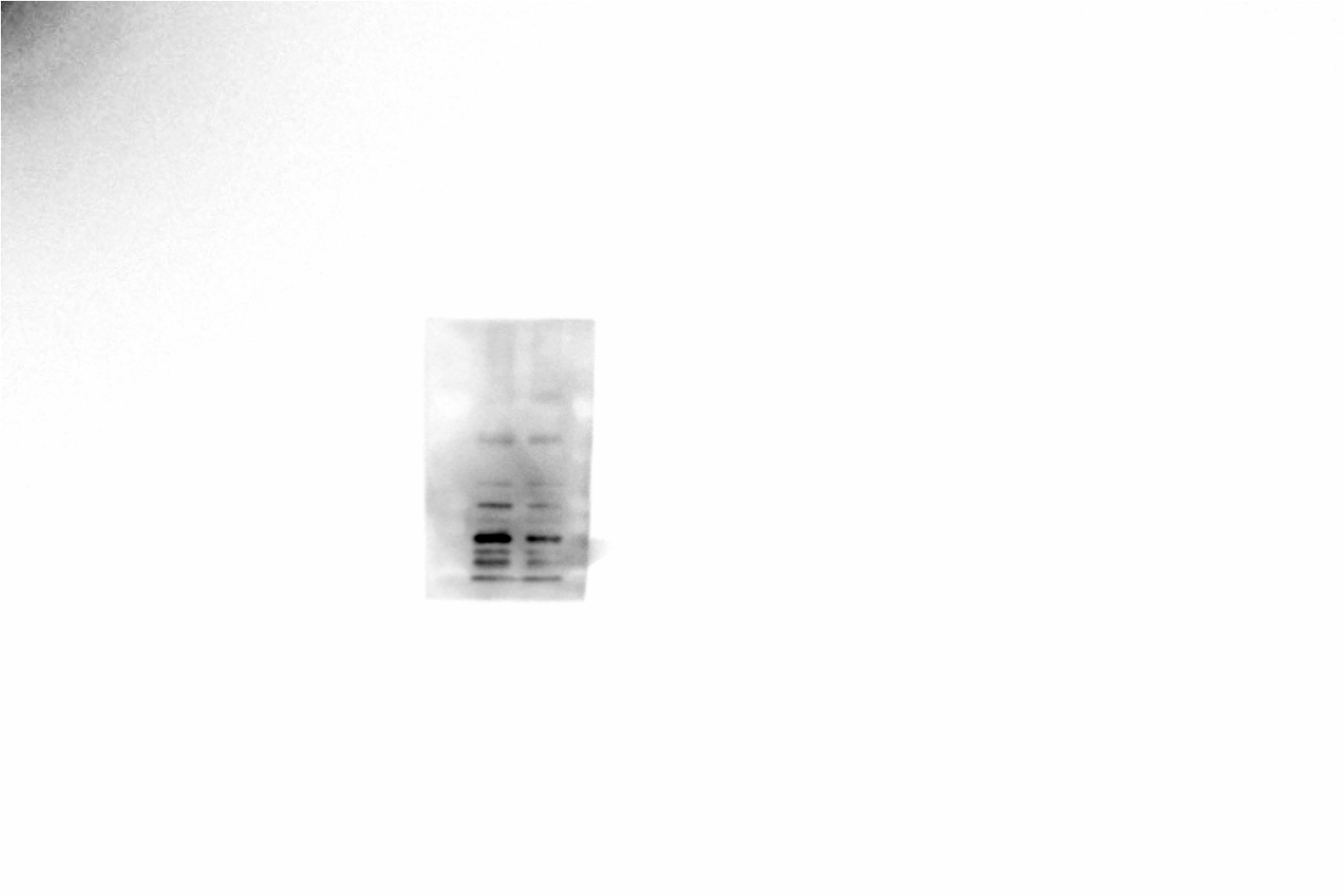


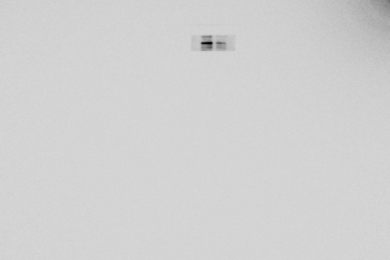

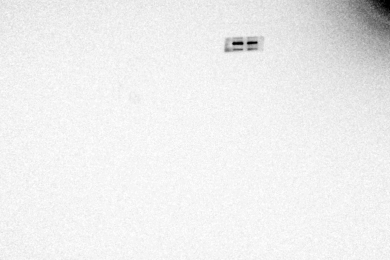

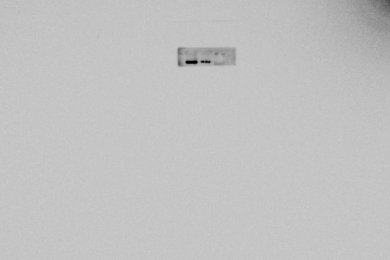


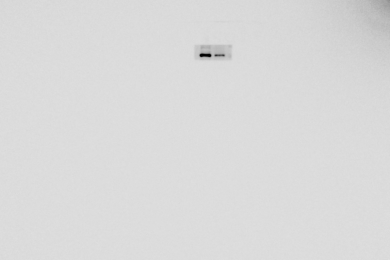

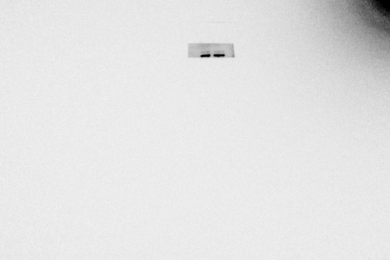

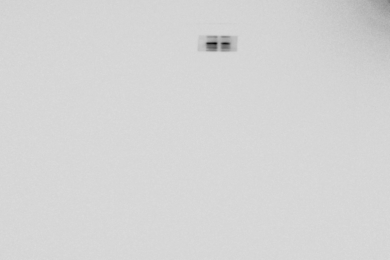


zoopery

NC shFGFR1 FGFR1





FGFR1

NC shFGFR1 FGFR1





LDHA

NC shFGFR1 FGFR1





LDHB

NC shFGFR1 FGFR1





H3K18la

NC shFGFR1 FGFR1

Pan Kla

NC shFGFR1 FGFR1

GAPDH

Downstream Gene Verification

GAPDH HDAC2 HK2

PFKM SIRT3

GAPDH HDAC2 HK2

PFKM SIRT3

GAPDH HDAC2 HK2

PFKM SIRT3

CO-IP

IB:

FGFR1 SIRT3

Input:

GAPDH FGFR1 SIRT3

Western blot detects upstream and downstream gene expression

FGFR1 SIRT3 GAPDH

Lactase and cyclin testing

GAPDH LDHA LDHB

CCNE1 CCND1

CHX

GAPDH FGFR1 SIRT3

MG132

GAPDH FGFR1 SIRT3
